# Europium–Magnesium–Aluminum-Based Mixed-Metal Oxides as Highly Active Methane Oxychlorination Catalysts

**DOI:** 10.1021/acscatal.2c06344

**Published:** 2023-03-30

**Authors:** Bas Terlingen, Jelle W. Bos, Mathieu Ahr, Matteo Monai, Coert van Lare, Bert M. Weckhuysen

**Affiliations:** † Inorganic Chemistry and Catalysis group, Debye Institute for Nanomaterials Science and Institute for Sustainable and Circular Chemistry, Utrecht University, Universiteitsweg 99, 3584 CG Utrecht, The Netherlands; ‡ Nobian, Zutphenseweg 10, 7418 AJ Deventer, The Netherlands

**Keywords:** methane activation, oxychlorination, operando spectroscopy, magnesium, aluminum, europium

## Abstract

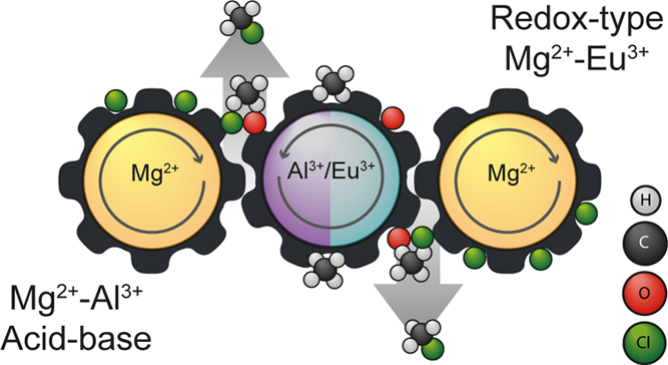

Methane oxychlorination (MOC) is a promising reaction for the production of liquefied methane derivatives. Even though catalyst design is still in its early stages, the general trend is that benchmark catalyst materials have a redox-active site, with, e.g., Cu^2+^, Ce^4+^, and Pd^2+^ as prominent showcase examples. However, with the identification of nonreducible LaOCl moiety as an active center for MOC, it was demonstrated that a redox-active couple is not a requirement to establish a high activity. In this work, we show that Mg^2+^–Al^3+^-based mixed-metal oxide (MMO) materials are highly active and stable MOC catalysts. The synergistic interaction between Mg^2+^ and Al^3+^ could be exploited due to the fact that a homogeneous distribution of the chemical elements was achieved. This interaction was found to be crucial for the unexpectedly high MOC activity, as reference MgO and γ-Al_2_O_3_ materials did not show any significant activity. Operando Raman spectroscopy revealed that Mg^2+^ acted as a chlorine buffer and subsequently as a chlorinating agent for Al^3+^, which was the active metal center in the methane activation step. The addition of the redox-active Eu^3+^ to the nonreducible Mg^2+^–Al^3+^ MMO catalyst enabled further tuning of the catalytic performance and made the EuMg_3_Al MMO catalyst one of the most active MOC catalyst materials reported so far. Combined operando Raman/luminescence spectroscopy revealed that the chlorination behavior of Mg^2+^ and Eu^3+^ was correlated, suggesting that Mg^2+^ also acted as a chlorinating agent for Eu^3+^. These results indicate that both redox activity and synergistic effects between Eu, Mg, and Al are required to obtain high catalytic performance. The importance of elemental synergy and redox properties is expected to be translatable to the oxychlorination of other hydrocarbons, such as light alkanes, due to large similarities in catalytic chemistry.

## 1 Introduction

Efficient conversion routes for the production of liquefied methane derivatives are pivotal for the feasibility of methane utilization.^1^ On-site production of methane-derived liquids would allow for cost-efficient transportation, which is currently one of the largest economic obstacles.^2,3^ The direct methane conversion routes, i.e., the oxidative coupling of CH_4_ (OCM), the CH_4_-to-methanol (MTM), the nonoxidative CH_4_ dehydroaromatization (MDA), and the (oxy)halogenation of CH_4_, are preferred over the indirect route as the energy-intensive endothermic methane reforming can be circumvented.^2,4−8^ The design criteria for an efficient methane conversion process consist of a high methane conversion at a high selectivity to the desired product, preferably at low energy consumption. The methane oxychlorination (MOC) reaction, which can be written as
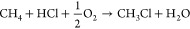
1has the potential to fulfill these design criteria.^9−17^

Several reported catalyst materials showed promising catalytic behavior in the MOC reaction.^9,11,13,18^ High conversion levels of methane and good selectivity to the desired CH_3_Cl reaction product are achieved at a relatively mild temperature and pressure for benchmark catalysts. Even though the development of MOC catalysts is still in an early phase, a redox-active center apparently seems essential for enhanced catalytic performance in MOC chemistry. The readily reducible elements Cu^2+^, Ce^4+^, and Pd^2+^ all show great activity in the MOC reaction.^11,13,18−20^ Even for the less reducible Eu^3+^, the redox properties are likely to play a significant role in the reaction mechanism.^21^

With the MOC field focusing on redox-active elements, catalyst compositions based on nonreducible elements are being somewhat overlooked. However, the nonreducible LaOCl was shown to be an active catalyst in the reaction, demonstrating that a stable redox-active couple is not essential to obtain high MOC activity.^14−17^ Interestingly, LaCl_3_ can even realize a chlorine atom exchange reaction between CH_2_Cl_2_ and CCl_4_.^22^ Moreover, La^3+^ was shown to act as a chlorine reservoir when combined with Eu^3+^ in mixed-metal oxide (MMO) catalysts, leading to higher MOC rates due to the synergy between the two elements.^23^

Inspired by these findings, we show in this work that very active MOC catalysts can be obtained by the combination of nonreducible and relatively inactive MgO and γ-Al_2_O_3_ in MMO catalyst materials. Stable performance for the MOC reaction was observed over a 100 h period, revealing that long-term stability could be achieved. While both Mg^2+^ and Al^3+^ have been successfully applied in/as catalysts for reactions involving halogens, including olefin polymerization catalysis,^10,13,24−31^ to the best of our knowledge, this is the first record of their application for MOC.

Considering that the MgO and γ-Al_2_O_3_ reference materials did not show significant MOC performance, the exceptionally high activity of the Mg^2+^–Al^3+^ MMO was highly unexpected. Operando Raman spectroscopy was used to understand the reason for the observed synergy. We found that Mg^2+^ acted as a chlorine buffer, providing chlorine for methane activation on Al^3+^, which would otherwise be poorly chlorinated.

Despite their very good MOC activity, Mg^2+^–Al^3+^ MMO showed a somewhat poor selectivity toward CH_3_Cl compared to the state-of-the-art. Selectivity was also not tunable by varying the HCl concentration, as is the case for redox-active catalysts.^18,23^ To overcome this issue, we introduced various amounts of redox-active Eu^3+^ in the Mg^2+^–Al^3+^ MMO by partial substitution of Al^3+^ in the layered double hydroxide (LDH) precursor, without altering the MMO crystal structure and surface area. The introduction of the redox-active site significantly improved the catalytic performance of the MMO materials in the MOC reaction, in terms of both activity and selectivity.

## 2 Experimental Section

### 2.1 Catalyst Synthesis

The MMO catalysts studied herein were prepared starting from layered double hydroxide (LDH) precursor materials, synthesized by the coprecipitation method.^32−34^ A round-bottom flask was filled with 50 mL of demineralized water, and the pH was adjusted to a value of 10 by the addition of a 1 M solution of Na_2_CO_3_·10 H_2_O (>99%, Sigma-Aldrich) in demineralized water. The metal chloride salts, i.e., MgCl_2_·6H_2_O (>99%, Acros Organics), AlCl_3_·6H_2_O (99%, Acros Organics), and EuCl_3_·*x*H_2_O (>99,9%, Alfa Aesar), were dissolved in 18 mL of demineralized water and loaded in a syringe. The amount of metal chloride salts was calculated to yield 1 g of MMO per batch. Next, the metal chloride salt solution was added to the round-bottom flask at a rate of 1 mL/min and the pH was kept between 9.9 and 10.1 by adding 18 mL of 1 M Na_2_CO_3_, immediately forming white precipitates. Once the Na_2_CO_3_ solution had been added, the pH was corrected by adding a 1 M solution of NaOH (pellets, 98%, Alfa Aesar) in demineralized water. Next, the precipitates were aged for 20 h at 75 °C before being filtrated and washed 3 times with demineralized water. Finally, the LDH materials were dried at 120 °C for 3 h and finally calcined at 450 °C in static air for 8 h to yield the MMO.

The ratio of Eu^3+^/Mg^2+^/Al^3+^ was calculated to yield the following final MMO’s/Mg_2_AlO_3.5_, Mg_3_AlO_4.5_, Mg_4_AlO_5.5_, Eu_0.06_Mg_2_Al_0.94_O_3.5_, Eu_0.08_Mg_3_Al_0.92_O_4.5_, and Eu_0.10_Mg_4_Al_0.90_O_5.5_ to obtain catalyst materials with a Mg^2+^/Al^3+^ ratio of 2, 3, or 4 with a Eu^3+^ concentration of 2 atom %. Reference MgO, Eu_0.16_Mg_3_Al_0.84_O_4.5_, and Mg_3_EuO_4.5_ were prepared according to the same procedure. γ-Al_2_O_3_ (high-surface-area catalyst support, Alfa Aesar), CeO_2_ (nanopowder, <50 nm particle size, 99,95%, Sigma-Aldrich), and MgAl_2_O_4_ (Spinel, <50 nm particle size, Sigma-Aldrich) were used in the catalytic tests without any pretreatment or modification. Eu/Mg_3_Al MMO and Eu/γ-Al_2_O_3_ were prepared by incipient wetness impregnation. The support material was impregnated with a europium(III) chloride hydrate (EuCl_3_·*x*H_2_O, Alfa Aesar, >99,9%) in demineralized water solution at room temperature under vacuum. The impregnated support was dried for 4 h at 80 °C under vacuum. Lastly, the dried impregnated solids were calcined in a static oven at 550 °C for 3 h using a ramp rate of 5 °C/min.

### 2.2 Catalyst Characterization and Thermodynamic Calculations

X-ray diffraction (XRD) patterns were obtained with a Bruker-AXS D8 powder X-ray diffractometer in the Bragg–Brentano geometry, using Cu Kα_1,2_ = 1.54184 Å, operated at 40 kV. The measurements were carried out between 22 and 65° using a step size of 0.02° and a scan speed of 0.5 s, with a 2 mm slit for the source. N_2_ adsorption isotherms were measured at −196 °C on a Micromeritics TriStar II Plus instrument. Prior to all measurements, samples were dried at 300 °C in a flow of N_2_ for at least 16 h. Specific surface areas were calculated using the multipoint Brunauer–Emmett–Teller (BET) method (0.05 < *p*/*p*_0_ < 0.25). Pore volumes were calculated by the *t*-plot method; pore size distributions were obtained by the Barrett–Joyner–Halenda (BJH) analysis; the Harkins and Jura thickness model was applied for the *t*-plot and BJH methods. Inductively coupled plasma-optical emission spectroscopy (ICP-OES) was applied to determine the chemical composition of the catalyst materials, using a SPECTRO CIROS^CCD^ instrument. ICP-OES samples were prepared by dissolving the solids in aqua regia. High-resolution transmission electron microscopy (HR-TEM) and high-angle annular dark-field scanning transmission electron microscopy (HAADF-STEM) were performed on a Talos F200× microscope equipped with four in-column SDD Super-X detectors to perform the energy-dispersive X-ray spectroscopy (EDS) analysis.

Thermodynamic calculations were performed with HSC 9 software using the equilibrium composition package from 100 to 1000 °C with a step size of 10 K. The inputs for the reaction equations are depicted in the corresponding figures.

### 2.3 Catalyst Testing

All of the catalytic tests and operando spectroscopy characterization experiments were performed in a lab-scale continuous-flow fixed-bed quartz reactor. Details on the experimental setup are reported elsewhere,^9^ and experimental definitions and calculations can be found in the Supporting Information Section 1.

For the methane oxychlorination (MOC) reaction, 100 mg of catalyst (125–425 μm sieve fraction) was loaded in a quartz reactor and heated to 350 °C and the desired feed mixture (i.e., CH_4_/HCl/O_2_/N_2_/He of 2:2:1:1:14 or 2:16:1:1:0 in mL/min) was fed into the reactor. A stabilization period of 15 min was applied, and then the ramp experiment of 1 °C/min was commenced to 550 °C. For the stability tests, 250 mg of catalyst (125–425 μm sieve fraction) was loaded in a quartz reactor and heated to 450 °C under N_2_ with a 10 °C/min heating rate. The feed was adjusted to CH_4_/HCl/O_2_/N_2_:He of 2:2:1:1:14 (in mL/min), and the experiment was performed for 100 h. For the determination of the activity–selectivity relation, 100 mg of catalyst (125–425 μm sieve fraction) was loaded in a quartz reactor to 350 °C under N_2_ with a 10 °C/min heating rate. The catalyst was subjected to CH_4_/HCl/O_2_/N_2_/He of 2:2:1:1:14 (in mL/min) for 45 min. The temperature was increased to 550 °C with increments of 10 °C with a heating rate of 5 °C/min and kept at every temperature step for 45 min to obtain the steady-state activity. The subsequent chlorination–dechlorination–oxychlorination experiments were carried out at 500 °C. The applied CH_4_/HCl/O_2_/N_2_/He ratio was 0:20:0:20:0 (chlorination step, in mL/min), 2:0:1:1:16 (dechlorination step, in mL/min), or 2:1:1:1:14 (oxychlorination step, in mL/min).

For the HCl oxidation reaction (4 HCl + O_2_ → 2 Cl_2_ + 2 H_2_O), 100 mg of catalyst (125–425 μm sieve fraction) was loaded in a quartz reactor. Temperature ramp experiments were performed from 350 to 550 °C at a ramp rate of 1 °C/min under the desired feed mixture (i.e., CH_4_/HCl/O_2_/N_2_/He of 0:2:1:1:16 or 0:16:1:1:2 in mL/min).

## 3 Results and Discussion

### 3.1 Catalyst Material Synthesis and Characterization

**Figure 1:**
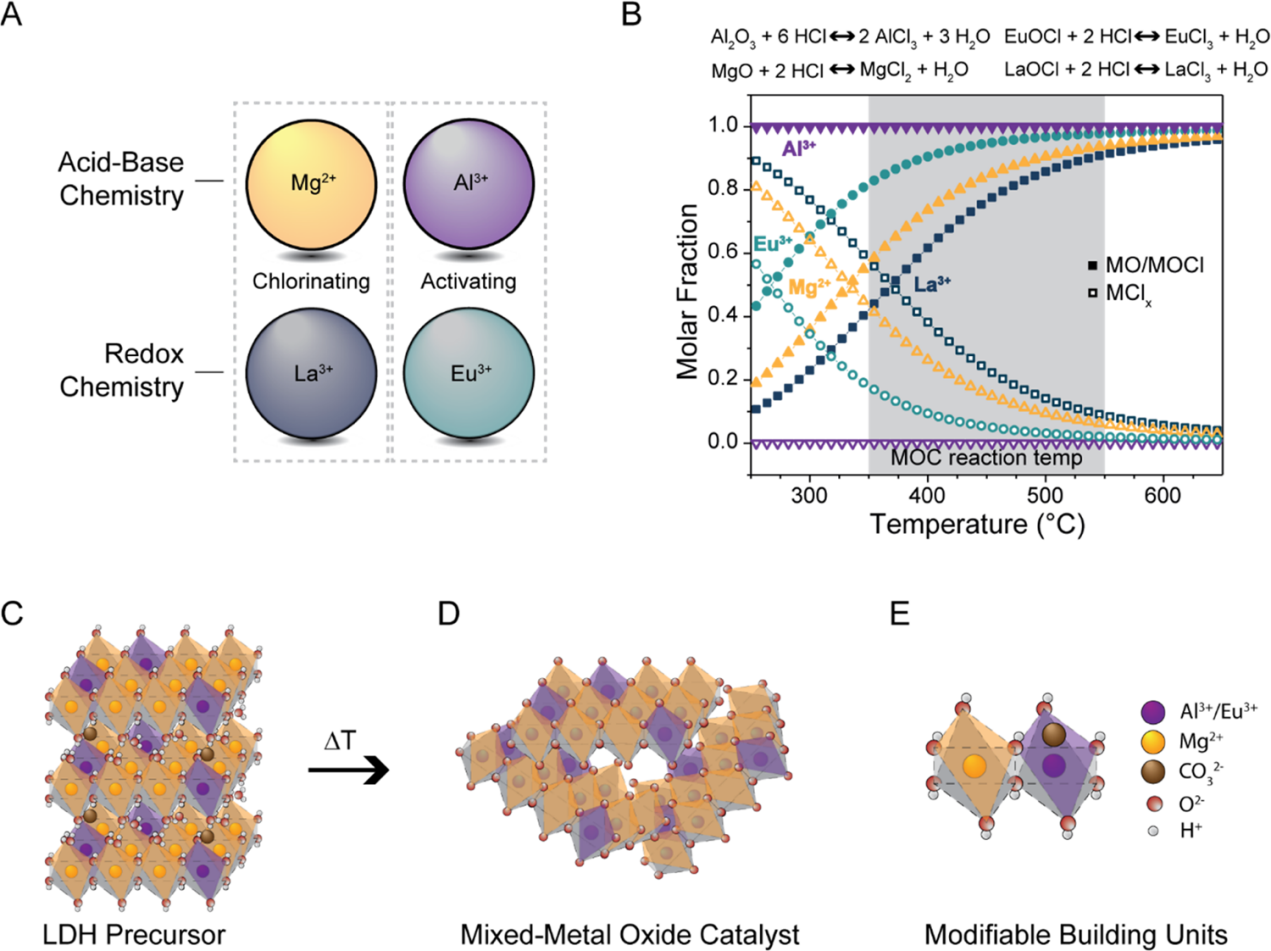
Concept and experimental approach applied for the design of active MOC catalysts. (A) The synergistic concept reported for redox-active La^3+^–Eu^3+^, where La^3+^ acted as a chlorinating agent and Eu^3+^ as an active element, was adapted for catalyst materials based on nonreducible Mg^2+^ and Al^3+^. (B) Thermodynamic equilibrium calculations for the chlorination of the studied metal oxides and oxychlorides, performed with the HSC Chemistry 9.1 program, showing that Mg^2+^ can fulfill a similar role as La^3+^ as a chlorine buffer, while both Al^3+^ and Eu^3+^ are not easily chlorinated. (C–E) The synthesis approach applied here exploits the tunable character of the layered double hydroxide (LDH) composition to yield the mixed-metal oxide (MMO) catalyst material to obtain a homogeneous distribution of chemical elements. (E) The octahedral building units that compose the LDH can be readily modified without drastically changing the MMO physicochemical properties. This approach allows for a fair comparison of different catalyst compositions.

In this work, the synergistic concept reported for the La^3+^–Eu^3+^ system in the methane oxychlorination (MOC) reaction, where La^3+^ acted as a chlorinating agent and Eu^3+^ as an active element, was translated to the Mg^2+^–Al^3+^ system (Figure 1A).^23^ Thermodynamic calculations for metal oxide or oxychloride chlorination evidenced the chemical similarities between Mg^2+^ and La^3+^ as their equilibrium compositions in the presence of HCl composed of roughly the same metal oxide/chloride ratio (Figure 1B). On the other hand, both Eu^3+^ and Al^3+^ are difficult to chlorinate under reaction conditions (Figure 1B), suggesting that a chlorine buffer may be beneficial for Al^3+^ in a similar way as for Eu^3+^. However, one significant difference with the La^3+^–Eu^3+^ system is that both Mg^2+^ and Al^3+^ are not reducible under the applied reaction conditions of the MOC reaction. Hence, any observed activity relies on the acid–base properties of the material, as redox-type chemistry can be excluded.

To exploit the mutual interaction between Mg^2+^ and Al^3+^, an intimate contact between the chemical elements is desired. To achieve this goal, we followed an approach based on the synthesis of layered double hydroxides (LDHs)^32−41^ (Figure 1C), followed by a calcination step to obtain mixed-metal oxide (MMO)^42−44^ catalyst materials (Figure 1D). The modifiable building units (Figure 1E) further allowed for the facile addition of the redox-active Eu^3+^, thereby opening the opportunity to functionalize the material with redox properties.

Accordingly, MMO and Eu-MMO catalysts were prepared by coprecipitation, and we refer to Section 2.1 for details on the synthesis procedure. The MMO catalyst materials are based on nonreducible Mg^2+^ and Al^3+^, i.e., Mg_2_AlO_3.5_, Mg_3_AlO_4.5_, and Mg_4_AlO_5.5_ will be further denoted as Mg_2_Al, Mg_3_Al, and Mg_4_Al MMOs, respectively. The Eu-MMO catalyst materials, for which Al^3+^ was partially substituted for the reducible Eu^3+^, i.e., Eu_0.06_Mg_2_Al_0.94_O_3.5_, Eu_0.08_Mg_3_Al_0.92_O_4.5_, and Eu_0.10_Mg_4_Al_0.90_O_5.5_, will be further denoted in the form of EuMg_2_Al, EuMg_3_Al, and EuMg_4_Al MMOs, respectively. An overview of the catalyst compositions and their corresponding physicochemical properties is provided in Table 1.

**Table 1:** Overview of the Physicochemical Properties of the Mixed-Metal Oxide (MMO) and Eu-MMO Catalyst Materials Prepared by Coprecipitation^a^ For All Catalyst Materials, the BET Surface Area (*S*_BET_), Pore Volume (*V*_Pore_), the Lattice Parameter a as Determined with X-ray Diffraction (XRD), and the Molar Ratio as Determined with Inductively Coupled Plasma-Optical Emission Spectroscopy (ICP-OES) Are Given

		N_2_ physisorption			lattice parameter
catalyst material	further denoted as	*S*_BET_ (m^2^/g)	*V*_pore_ (cm^3^/g)	(Eu^3+^/)Mg^2+^/Al^3+^ molar ratio (ICP-OES)	*a* (Å)^b^
Mg_2_AlO_3.5_	Mg_2_Al	212.1	0.74	2:0.95	4.168
Mg_3_AlO_4.5_	Mg_3_Al	257.8	1.27	3:0.96	4.183
Mg_4_AlO_5.5_	Mg_4_Al	229.6	0.98	4:0.95	4.194
Eu_0.06_Mg_2_Al_0.94_O_3.5_	EuMg_2_Al	221.9	0.92	0.06:2:0.89	4.172
Eu_0.08_Mg_3_Al_0.92_O_4.5_	EuMg_3_Al	275.0	1.53	0.08:3:0.90	4.195
Eu_0.10_Mg_4_Al_0.90_O_5.5_	EuMg_4_Al	206.2	0.50	0.10:4:0.9	4.191

a For all catalyst materials, the BET surface area (*S*_BET_), pore volume (*V*_Pore_), the lattice parameter a as determined by X-ray diffraction (XRD), and the molar ratio as determined by inductively coupled plasma-optical emission spectroscopy (ICP-OES) are given

b The (200) reflection was used for determining the lattice parameter *a*; see the Supporting Information Section 1.

**Figure 2:**
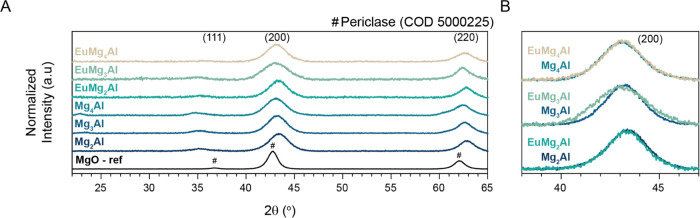
(A) X-ray diffraction (XRD) patterns of the as-synthesized MMO catalyst materials. The reflections of the MMO materials are indicated in the graph. (B) A zoom-in of the (200), where the diffractions are ordered based on the Mg^2+^/M^3+^ ratio where the Eu-MMO is plotted together with the corresponding MMO.

The Brunauer–Emmett–Teller (BET) surface area (*S*_BET_) and pore volume (*V*_pore_) of the MMO materials were quite comparable with *S*_BET_ values between 206.2 and 275.0 m^2^/g, while the *V*_pore_ ranged between 0.50 and 1.53 cm^3^/g. The catalyst with a Mg^2+^/Al^3+^ ratio of 3 gave the highest *S*_BET_ and *V*_pore_ for both sets of MMO catalysts. In all cases, phase-pure MMO materials were synthesized, as shown by the characteristic defective MgO periclase reflections (COD 5000225), i.e., (111), (200), and (220) (Figure 2A). A shift to lower angles in the X-ray diffraction (XRD) was observed with an increasing Mg^2+^/Al^3+^ ratio (Figure 2B), coinciding with the increase of the lattice parameter a (Table 1), as a higher ratio of the larger Mg^2+^ is incorporated into the crystal structure.^45^ No lattice expansion was observable when comparing the MMO catalysts with and without Eu^3+^ (Figure 2B). However, no XRD reflections corresponding to segregated Eu^3+^, Mg^2+^, and or Al^3+^ phases were observed. Also, no extra-framework phases were observed with elemental mapping, as revealed with energy-dispersive X-ray spectroscopy (EDS) analysis (Figure S2). Hence, we can conclude that Eu^3+^ is highly dispersed throughout the material, and we can exclude the incorporation of Eu^3+^ into the crystal structure. Importantly, the similar physicochemical properties observed for the studied MMO catalysts enabled us to rationalize their catalytic activity mainly in terms of composition.

The synthesis procedure yielded the desired molar ratio for all catalyst materials, as evidenced by inductively coupled plasma-optical emission spectroscopy (ICP-OES) (Table 1). A theoretical Eu^3+^ concentration of 2 atom % was used, as a higher lanthanide concentration yielded extra-framework phases due to a mismatch in ionic radii and charge (Figure S1).^34^ Since extra-framework phases would have added another degree of complexity when trying to study these catalyst materials, the Eu^3+^ content was kept at 2 atom %, enabling a fair comparison of the Mg^2+^/Al^3+^ ratio and the effect of Eu^3+^.

### 3.2 Methane Oxychlorination over Mg^2+^–Al^3+^ Mixed-Metal Oxides

**Figure 3:**
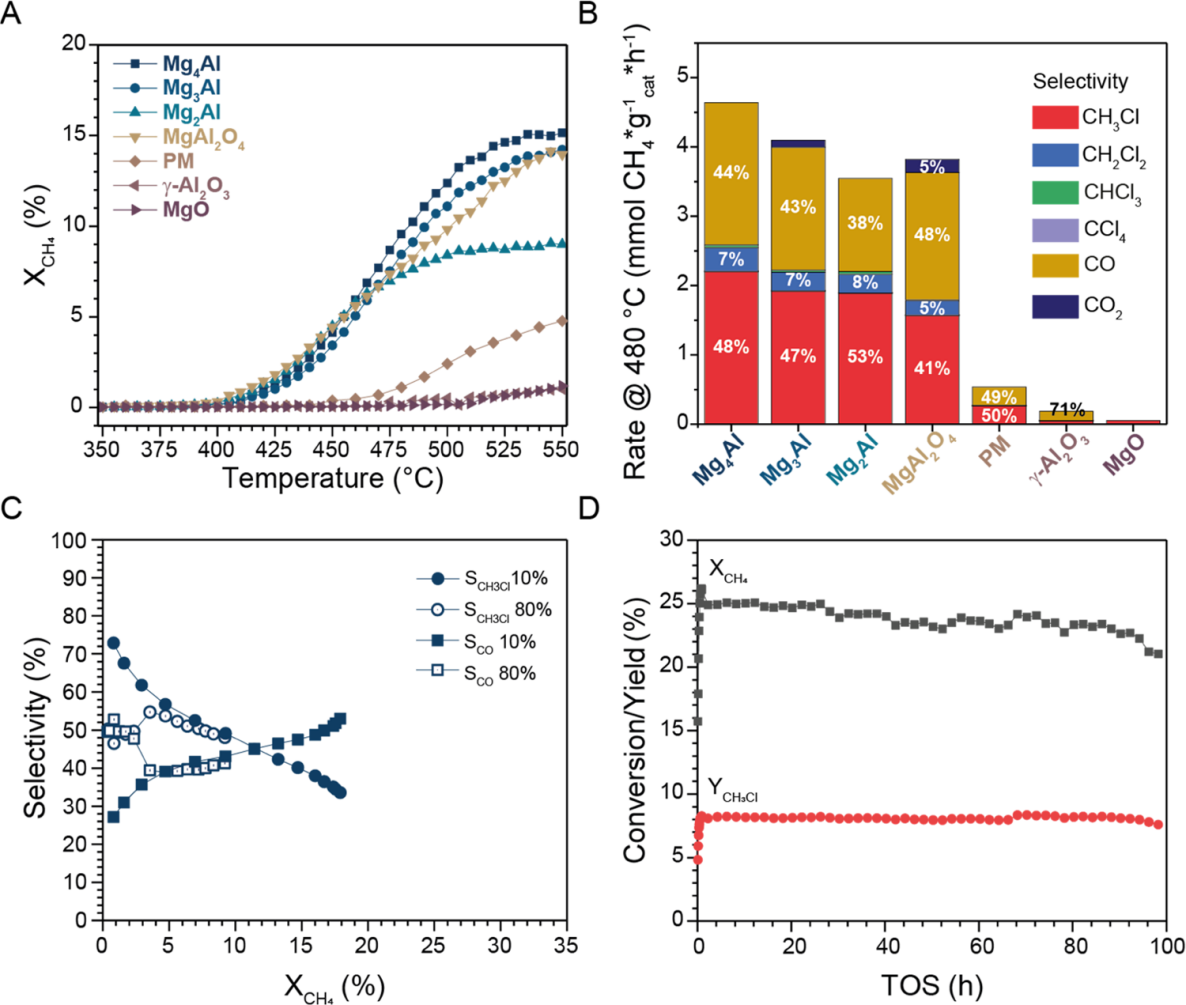
Overview of the catalytic performance of Mg*_x_*Al mixed-metal oxide (MMO) catalyst materials and reference materials in the methane oxychlorination (MOC) reaction. A physical mixture with a 3:1 ratio of MgO/γ-Al_2_O_3_ was also tested, denoted as PM. (A) The CH_4_ conversion (*X*_CH_4__) plotted vs the reaction temperature under 10 vol % of HCl in the feed. (B) The CH_4_ conversion rate normalized to the amount of catalyst at 480 °C under 10% HCl. The selectivity at 480 °C is indicated in the bar. (C) The nonisothermal activity plotted versus the CO selectivity (*S*_CO_, square) and the CH_3_Cl selectivity (*S*_CH_3_Cl_, circle) under 10% (filled) and 80% (open) HCl in the feed. (D) Stability tests for the Mg_4_Al MMO catalyst over 100 h of reaction time-on-stream (TOS) at 480 °C, showing only slight deactivation in terms of *X*_CH_4__ and CH_3_Cl yields (Y_CH_3_Cl_).

The Mg^2+^–Al^3+^ MMO catalyst materials were all very active for the MOC reaction (Figure 3A). Since the reference MgO and γ-Al_2_O_3_ did not show any significant MOC activity (Figure 3A), the synergistic interaction between Mg^2+^ and Al^3+^ appears to be the main reason for the observed activity in the Mg^2+^–Al^3+^ MMO. When normalized by surface area, a physical mixture of MgO and γ-Al_2_O_3_ with a Mg^2+^/Al^3+^ ratio of 3 (denoted as PM in Figure 3) was found to be at least 5.5 times less active than any of the MMOs (Figure S3), indicating that an intimate contact between Mg^2+^ and Al^3+^ is needed to obtain high methane conversion (*X*_CH_4__). This observation is further strengthened by the fact that 2 wt % of Mg/SiO_2_ and Mg/γ-Al_2_O_3_ did not reveal any significant activity (*X*_CH_4__ < 1% at 480 °C, results not shown). Accordingly, a commercial MgAl_2_O_4_ reference, where Mg and Al are intimately mixed, performed similarly to the synthesized MMO (Figure 3A).

Despite the similar general trends observed for MMO activity, the conversion curve of Mg_2_Al MMO differed quite significantly from the other Mg*_x_*Al MMO catalysts in the region above 475 °C. The activity of Mg_2_Al MMO leveled off to a final value of 9%, while for Mg_3_Al, Mg_4_Al, and MgAl_2_O_4_ MMOs, the *X*_CH_4__ increased steadily up to a temperature of 510 °C after which it leveled off to 15%. Further research is needed to clarify the nature of the lower activity of Mg_2_Al compared to that of Mg_3_Al and Mg_4_Al MMOs at elevated temperatures.

The highest activity was observed for the Mg_4_Al MMO catalyst material, which had a methane conversion rate of 4.63 mmol·g_cat_^–1^·h^–1^ at 480 °C, though the activities of Mg*_x_*Al MMO and MgAl_2_O_4_ were all in the same range (Figure 3B). These activity values, when normalized by catalyst weight, are higher than those reported for most MOC catalyst materials, which can be partially ascribed to the low density of the MMO (ρ_sieve,MMO_ ≈ 0.67 g/cm^3^ vs ρ_sieve,EuOCl_ ≈ 1.50 g/cm^3^). Nevertheless, a poor selectivity was observed for all Mg*_x_*Al MMO catalysts at 480 °C (Figure 3B). This is further evidenced by the activity–selectivity plot, where a maximum CH_3_Cl selectivity (*S*_CH_3_Cl_) of 70% at *X*_CH_4__ <1% under 10% HCl was obtained for Mg_3_Al MMO (Figure 3C, filled symbols). This performance does not meet the standard that is reported in the literature where *S*_CH_3_Cl_ > 70% at *X*_CH_4__ > 10% is typically achieved.^11,13,23,46^ A large part of the methane is converted to CO, which was the dominant product at temperatures above 490 °C.

A reported strategy to minimize the CO selectivity applied for EuOCl was to enhance the HCl concentration in the feed.^9,23^ Here, the same strategy did not result in an improved activity/selectivity relation for Mg_3_Al MMO (Figure 3C, open symbols). Conversely, increasing the HCl concentration from 10 to 80% resulted in a drop in activity from 4.08 to 1.17 mmol·g_cat_^–1^·h^–1^ at 480 °C for Mg_3_Al, a trend that is observable for the entire tested temperature range for all Mg^2+^–Al^3+^ catalyst materials. The reason for this deactivation under increased HCl concentrations in the feed is further investigated in Section 3.4. Lastly, the Mg_4_Al MMO catalyst showed a slight decrease in *X*_CH_4__ over a 100 h time-on-stream (TOS) (Figures 3D and S4A) after an initial induction period. This indicates that the Mg^2+^–Al^3+^-based MMO catalysts could possess long-term stability in the MOC reaction. The nature of this induction period can be explained by the fact that no steady-state catalyst chlorination was reached, which is pivotal for steady-state performance (see Section 3.4).

### 3.3 Methane Oxychlorination over Eu^3+^–Mg^2+^–Al^3+^ Mixed-Metal Oxides

Even though the nonreducible Mg*_x_*Al MMO materials were active and showed stable performance, a good selectivity in the reaction is crucial for the eventual application. The addition of a redox-active element could further improve the catalytic properties and enable the tuning of the activity–selectivity relation. The catalyst synthesis procedure of the MMO materials allowed for the facile partial replacement of Al^3+^ by the redox-active Eu^3+^ without altering the crystal structure and *S*_BET_/*V*_pore_ (Table 1 and Figure 2A) and yielding a high dispersion of Eu^3+^ throughout the material. The choice for Eu^3+^ was made as the working principles of Eu^3+^ in the MOC reactions are relatively well studied. Typically, increasing the HCl concentration in the feed leads to a lower CO selectivity for Eu^3+^-based catalysts, even at increased conversion levels.^9,23^ The Mg*_x_*Al MMO catalyst itself performed poorly under such conditions in terms of *S*_CO_ and conversion rate. Thus, the functionalization of the Mg*_x_*Al MMO with Eu^3+^ could add the functionalities, which the Mg*_x_*Al MMO lacked.

**Figure 4:**
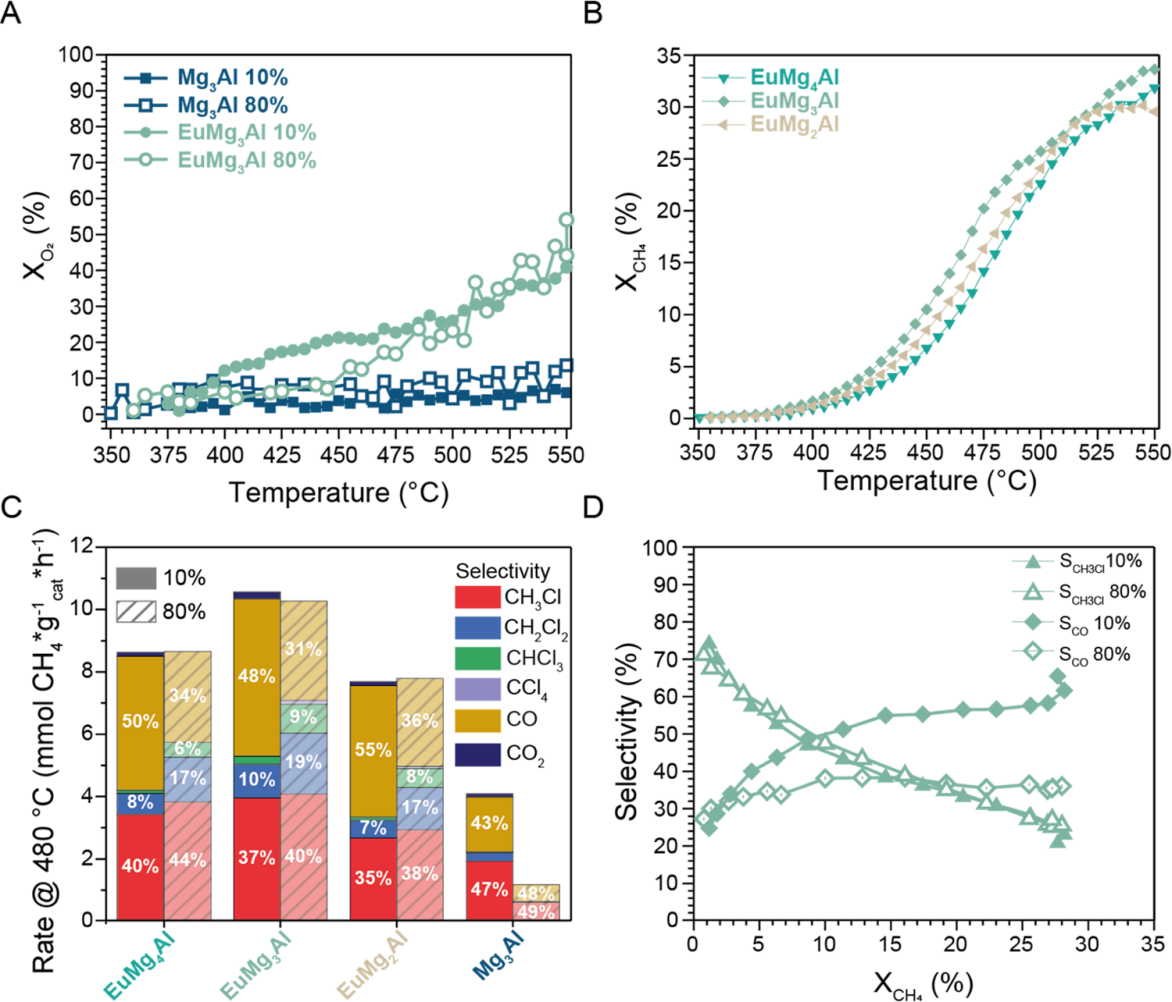
Overview of the catalytic performance of EuMg*_x_*Al mixed-metal oxide (MMO) catalyst materials in the methane oxychlorination (MOC) reaction. (A) The O_2_ conversion (*X*_O_2__) plotted vs the temperature for the HCl oxidation under 10 and 80% HCl over Mg_3_Al and EuMg_3_Al MMOs. (B) The CH_4_ conversion (*X*_CH_4__) plotted vs the temperature under 10% HCl in the feed. (C) The CH_4_ conversion rate normalized to the amount of catalyst at 480 °C under 10 and 80% HCl in the feed. The selectivity at 480 °C is indicated in the bar. (D) The nonisothermal activity plotted vs the CO selectivity (*S*_CO_, diamond) and the CH_3_Cl selectivity (*S*_CH_3_Cl_, triangle) under 10% (filled) and 80% (open) HCl in the feed.

First of all, the 100 h stability test revealed that activity was largely preserved by the addition of Eu^3+^ (Figure S4B). The postcharacterization with the XRD of the catalyst after 24 h of reaction under the same conditions revealed that the Mg^2+^ in the catalyst was partially chlorinated to MgCl_2_ (Figure S5A). No Eu^3+^- or Al^3+^-based phases could be identified. After dechlorination, most of the MgCl_2_ has been converted to MgO, although traces of MgCl_2_ appear to be present (Figure S5B). Further analysis of the chlorinated spent catalyst is hampered by the hygroscopic nature of the formed metal chlorides. To get insight into the catalyst morphology and elemental distribution in the spent catalyst, we performed a dechlorination step to remove any chlorides (Figure S5C) so the catalyst could be analyzed with STEM-EDS (Figure S5D,E). The elemental mapping did not show any severe agglomeration of Eu^3+^, Al^3+^, and Mg^2+^ after 24 h of reaction and a subsequent dechlorination step. It must be stated that the catalyst is altered by the dechlorination step and might not be representative of the catalyst under working conditions. The addition of the Eu^3+^ as redox functionality was reflected in the fact that the EuMg_3_Al MMO was able to perform the HCl oxidation reaction, i.e., 4 HCl + O_2_ → Cl_2_ + 2 H_2_O, where typically a redox couple is needed for the Mars–van Krevelen-type of reactions,^47^ while the Mg_3_Al MMO was not (Figure 4A). This indicates that the reaction over Mg_3_Al MMO is almost exclusively a surface-driven reaction as the evolution of Cl_2_ is very minimal. With the addition of Eu^3+^, the reaction can occur over or via a redox-active site, enabling the formation of (some) Cl_2_ during oxychlorination. The preparation of the Mg*_x_*Al MMO catalysts with only 2 atom % of Eu^3+^ greatly improved the catalytic activity in the MOC reaction. Generally speaking, the activity is doubled over the entire tested temperature range compared to the MMO materials (Figure 4B). All three EuMg*_x_*Al MMO catalysts behaved very similarly over the entire tested temperature range in terms of *X*_CH_4__, again revealing that the Mg/Al ratio did not influence the catalytic properties significantly. The high dispersion of Eu^3+^ in the Mg_3_Al MMO catalyst material was needed to observe enhancement, as at *T* = 550 °C the 2 wt % of Eu/γ-Al_2_O_3_ (*X*_CH_4__ = 14.7%) and 2 wt % of Eu/Mg_3_Al (*X*_CH_4__ = 17.2%), both prepared by impregnation, were only slightly more active compared to that of Mg_3_Al MMO (*X*_CH_4__ = 14.5%) (Figure S6). The highest activity was achieved for EuMg_3_Al MMO, possessing a methane conversion rate of 10.56 mmol·g_cat_^–1^·h^–1^ at 480 °C, a doubling compared to the Mg_3_Al MMO (Figure 4C). To put this conversion rate into a broader context, EuMg_3_Al MMO is one of the most active catalysts reported, higher than the reference CeO_2_ (8.0 mmol·g_cat_^–1^·h^–1^) and approaching FeO*_x_*–CeO_2_ with a methane conversion rate of roughly 15.7 mmol·g_cat_^–1^·h^–1^ under similar conditions.^13^ The functionalization of the Mg*_x_*Al MMO was further evidenced by the preservation of the activity under 80% HCl, conditions under which the Mg*_x_*Al MMO did not perform well. The addition of Eu^3+^ enhanced the methane conversion rate from 1.17 mmol·g_cat_^–1^·h^–1^ for Mg_3_Al MMO to 10.27 mmol·g_cat_^–1^·h^–1^ for EuMg_3_Al MMO, a 9-fold increment. The selectivity could be varied by altering the HCl concentration, as the *S*_CO_ is reduced for the Eu^3+^-MMO catalysts (Figure 4C). This was also evidenced by the activity–selectivity relation, where a reduction of the *S*_CO_ by as much as 30% point was observed when increasing the HCl concentration (Figure 4D). However, the *S*_CH_3_Cl_ did not respond to the varying HCl concentration as the 10 and 80% are almost exact overlays.

**Figure 5:**
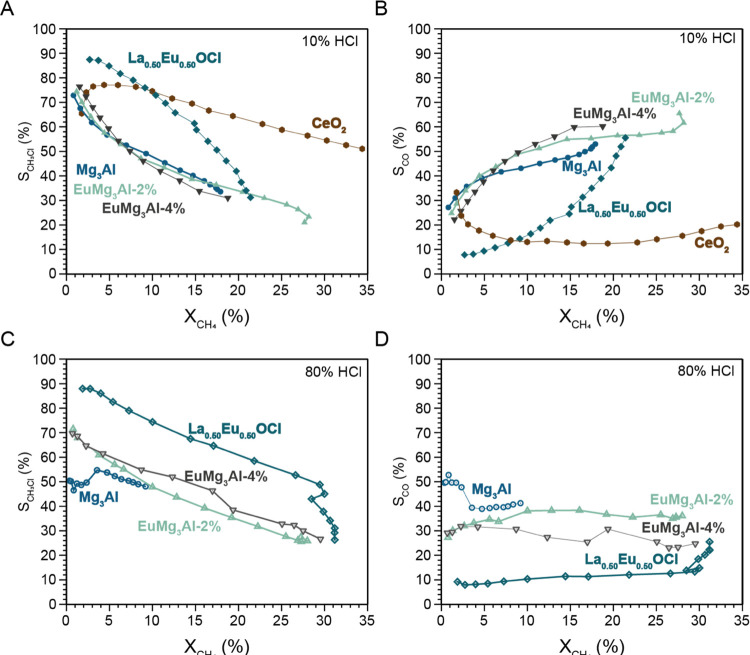
Nonisothermal activity–selectivity (*X*–*S*) relation for the methane oxychlorination (MOC) reaction plotted for Mg_3_Al, EuMg_3_Al-2%, EuMg_3_Al-4%, CeO_2_ benchmark, and La_0.50_Eu_0.50_OCl from ref (23) under (A, B) 10% HCl and (C, D) 80% HCl in the feed. The selectivity toward (A, C) CH_3_Cl and (B, D) CO is given. The *X*–*S* relation for CeO_2_ under 80% is not plotted due to low activity over the entire tested temperature range.

A plateau in the *S*_CO_ was observed from *X*_CH_4__ > 10% at a value of ∼35%. We hypothesize that this effect is caused by the fact that the Mg*_x_*Al MMO displays low activity under these conditions, suppressing the poorer *X*–*S* relation of Mg_3_Al, while Eu^3+^ becomes more active, resulting in improved catalytic performance. If this hypothesis is true, an increase in the Eu^3+^ content would further improve the *X*–*S* relation. Therefore, the nonisothermal *X*–*S* relation was also measured for the reference EuMg_3_Al MMO with 4 atom % of Eu^3+^ (Figure 5). For facile comparison, the *X*–*S* relation of the benchmark CeO_2_ and La_0.50_Eu_0.50_ is also plotted. The increase in the Eu^3+^ content from 2 to 4 atom % did not significantly improve the catalytic performance under 10% HCl. However, under 80% HCl, an increase in the *S*_CH_3_Cl_ was achieved, while a lowering of the *S*_CO_ was observed at the same time. The *S*_CH_3_Cl_ gradually decreased with increasing *X*_CH_4__ but the *S*_CO_ revealed a plateau between 23 and 30%. The catalytic performance positively correlated to the Eu^3+^ content in the catalyst, but the excellent *X*–*S* of La_0.50_Eu_0.50_OCl and CeO_2_ could not be matched.

**Figure 6:**
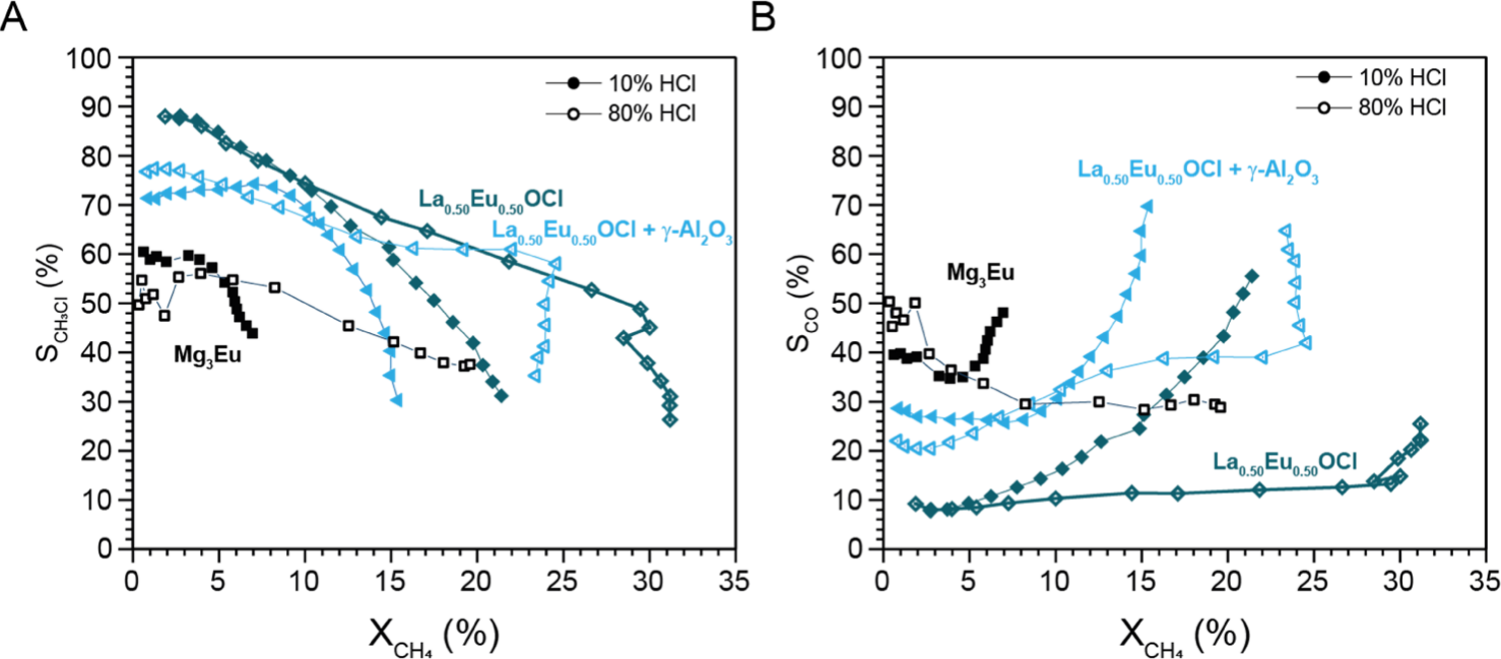
Nonisothermal activity–selectivity (*X*–*S*) relation for the methane oxychlorination (MOC) plotted for La_0.50_Eu_0.50_OCl from ref (23), La_0.50_Eu_0.50_OCl + γ-Al_2_O_3_, and Mg_3_Eu under 10% HCl (filled symbols) and 80% HCl (open symbols) in the feed. The La_0.50_Eu_0.50_OCl + γ-Al_2_O_3_ was a dual catalyst bed with 500 mg of La_0.50_Eu_0.50_OCl first contacting the gas feed and then 100 mg of γ-Al_2_O_3_ separated by quartz wool to prevent mixing. The selectivity toward (A) CH_3_Cl (*S*_CH_3_Cl_) and (B) CO (*S*_CO_) is given.

The CO formation was already quite pronounced at *X*_CH_4__ < 5% for Al^3+^-containing catalysts, and this proved difficult to circumvent by altering the reaction conditions and catalyst compositions (Figure S7). To show that Al^3+^ was the main contributor to the (unwanted) high CO selectivity, a dual catalyst bed was made. At the top, the gas stream first contacted the active La_0.50_Eu_0.50_OCl, producing a mix of chloromethanes. Subsequently, the product mix contacted 100 mg of γ-Al_2_O_3_ before being analyzed with GC (Figure 6). The *X*–*S* graphs show that the CO selectivity drastically deteriorated when compared to La_0.50_Eu_0.50_OCl.

Interestingly, the sum of *S*_CH_3_Cl_ and *S*_CO_ made up almost 100% of the total product formation over the entire *X*_CH_4__ range, revealing that the γ-Al_2_O_3_ catalytically destructed any formed polychlorinated C_1_ molecules to CO. This suggested that removing Al^3+^ from the formulation of the MMO might be beneficial, and accordingly, a Mg_3_Eu catalyst was prepared as a reference. However, the *X*_CH_4__ for the Mg_3_Eu catalyst was low, not exceeding 8%, with a minimum *S*_CO_ of 35%. The catalyst performed better under 80% HCl, where a minimum *S*_CO_ of 28% could be reached at the maximum *X*_CH_4__ of 20%. Nevertheless, the performance was unexpectedly low, possibly due to phase segregation leading to the suppression of synergy. Furthermore, the absence of Al^3+^ could also have profound effects on the structural stability of the material. Materials that are subjected to phase changes typically show a reduction of the available surface area, thereby lowering the activity.^15^

### 3.4 Operando Spectroscopy Study on Mg^2+^–Al^3+^ MMOs Revealed Synergy Mechanism

**Figure 7:**
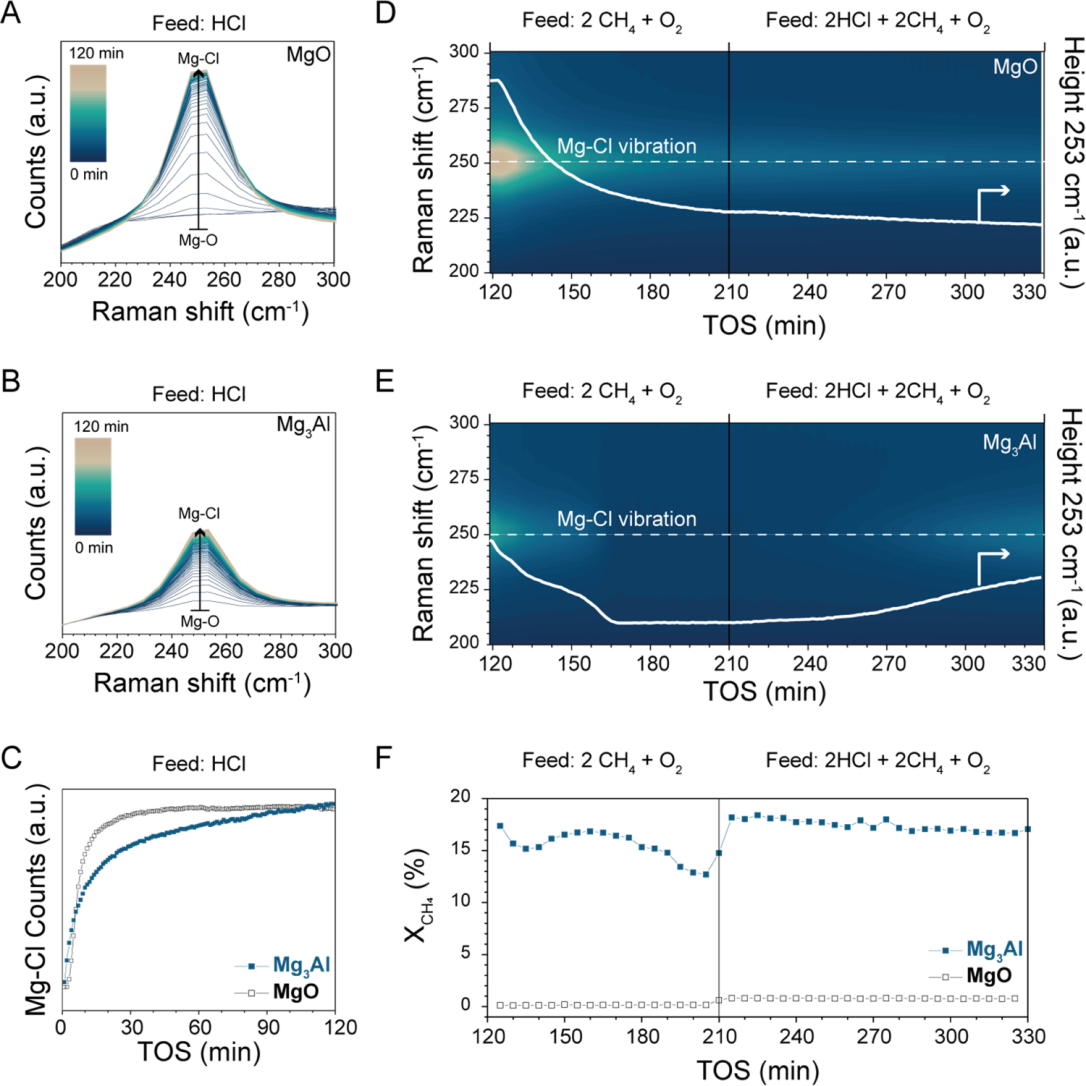
Operando Raman spectroscopy measurements performed during chlorination, dechlorination, and oxychlorination where the Mg^2^–Cl Raman stretching vibration is probed. (A, B) The Mg–Cl Raman vibration during the chlorination step is plotted for (A) MgO and (B) Mg_3_Al. (C) The height of the 253 cm^–1^ Mg–Cl peak plotted versus the time-on-stream (TOS) for both catalysts, showing a lower growth rate for Mg_3_Al. (D, E) The Mg–Cl Raman vibration is plotted versus the TOS during the dechlorination and oxychlorination steps for (D) MgO and (E) Mg_3_Al. Furthermore, the height profile of the Mg–Cl vibration at 253 cm^–1^ is given. Lastly, (F) methane conversion (*X*_CH_4__) was plotted versus the TOS during the dechlorination and oxychlorination steps for both catalysts.

To gain insights into the reaction over the Mg*_x_*Al MMOs, operando Raman spectroscopy was performed to study the (de)chlorination behavior of Mg^2+^ in MgO and Mg_3_Al MMO under varying reaction conditions (Figure 7). In the chlorination step, the catalysts were subjected to 50 v/v% HCl/N_2_, which caused the formation of MgCl_2_ in both cases, as indicated by the emergence of the Mg–Cl vibration at 253 cm^–1^ (Figure 7A,B). Important to note is that no other spectral changes were observed (Figure S8A). Any formation of AlCl_3_ would not be detectable with Raman due to its low boiling point of 180 °C. The formation of bulk AlCl_3_ was, however, excluded as γ-Al_2_O_3_ was proven stable under 24 h of oxychlorination conditions, as no weight loss, *S*_BET_ loss, phase change, or reactor staining was observed (see the Supporting Information Section 2.3). The final state was reached faster in the case of MgO compared to that of Mg_3_Al MMO (Figure 7C). We hypothesize that the chlorination of Mg^2+^ in Mg_3_Al MMO is more difficult due to the interaction with Al^3+^, as γ-Al_2_O_3_ is resistant to bulk chlorination under the applied conditions.

Subsequently, the catalysts were subjected to dechlorinating conditions to remove the chlorine by the reaction with methane and oxygen. In the case of MgO, partial dechlorination was observed, still preserving some of the Mg^2+^–Cl peaks (Figure 7D). At the same time, no products were formed (Figure 7F). Our hypothesis is that the dechlorination of MgCl_2_ to MgO is solely a thermodynamic effect as the thermodynamic equilibrium MgCl_2_/MgO ratio is pushed toward MgO with decreasing HCl present (Figure S8B). For Mg_3_Al MMO, however, the chlorine was completely removed from the catalyst, indicated by the complete disappearance of the Mg–Cl vibration (Figure 7E).

During the dechlorination step, significant *X*_CH_4__ was observed, indicating that chlorine was stored in the form of MgCl_2_ and released by the active Al^3+^. The activity gradually dropped as the Mg^2+^–Cl signal dropped as well, an indication that the chlorine stored in the catalyst was depleting. The fact that the catalyst was still active while the chlorine was depleted could be ascribed to the fact that the middle of the catalyst bed was probed while the bottom of the catalyst bed could still hold chlorine.

In the last step, MOC conditions were applied. The MgO catalyst did not show any significant spectral change. This could be due to the establishment of thermodynamic equilibrium between chlorinated and dechlorinated states or due to a kinetically limited steady state between chlorination and dechlorination. Since product formation was negligible, significant dechlorination due to reaction with methane can be excluded, suggesting that the thermodynamic equilibrium for (de)chlorination was reached. On the other hand, Mg^2+^ in Mg_3_Al MMO was partially chlorinated, and at the same time, a stable *X*_CH_4__ of ∼17% was observed. Based on our spectroscopic results, we propose an MOC reaction mechanism over Mg*_x_*Al MMO, in which the synergy between Mg^2+^ and Al^3+^ is rationalized. γ-Al_2_O_3_ was proven difficult to chlorinate (SI Section 2.3) and thus possessed low activity. Mg^2+^ was readily chlorinated by HCl and could be (partially) converted from MgO to MgCl_2_. However, MgCl_2_ was not able to efficiently catalyze the MOC reaction. The synergy occurred when chlorine could be stored by Mg and transferred to the Mg^2+^–Al^3+^ boundary. The available chlorine readily reacted away by Al^3+^ with CH_4_ and O_2_, enabling a catalytic cycle of chlorination and dechlorination when Mg^2+^–Al^3+^ can interact. In view of the nonreducibility of the two elements, we propose that the surface Mg–O–Al is chlorinated, which then follows a similar reaction mechanism as proposed for the nonreducible LaOCl.^17^

Lastly, operando Raman spectroscopy on Mg_3_Al MMO evidenced a correlation between the degree of Mg^2+^ chlorination and catalyst deactivation (Figure S9). Under 10% HCl (0–60 min), the chlorination rate and dechlorination of MgO moved to a steady state, where only partial Mg^2+^ chlorination was achieved. When the HCl was further increased to 20% (60–120 min), an acceleration in the Mg^2+^ chlorination rate was observed, coinciding with a dip in *X*_CH_4__. No sign of stabilization of the Mg–Cl Raman signal or the *X*_CH_4__ was apparent under 20% HCl. This deactivation/Mg^2+^ chlorination trend was continued until the final HCl concentration of 80% was fed (120–300 min). Thereafter, the HCl concentration in the feed was lowered, causing a partial and gradual dechlorination and at the same time a partial and sudden recovery of the activity. Hence, the catalytic performance of Mg_3_Al MMO was quite sensitive to an increase in HCl concentration, caused by catalyst over chlorination.

### 3.5 Combined Operando Luminescence and Raman Spectroscopy on the EuMg_3_Al Catalyst Material under MOC Conditions

For the EuMg_3_Al MMO catalyst, it was possible to analyze the chlorination–dechlorination behavior of Eu^3+^, alongside the chlorination–dechlorination behavior of MgO. This was done by detecting the luminescence signal of Eu^3+^, which is quenched at elevated temperatures (>300 °C) when Eu^3+^ is in the chlorinated state.^9,23^ The Raman vibration of Mg^2+^–Cl and the luminescence signal of the ^5^D_1_ → ^7^F_2_ transition were plotted during the chlorination, dechlorination, and oxychlorination steps.

**Figure 8:**
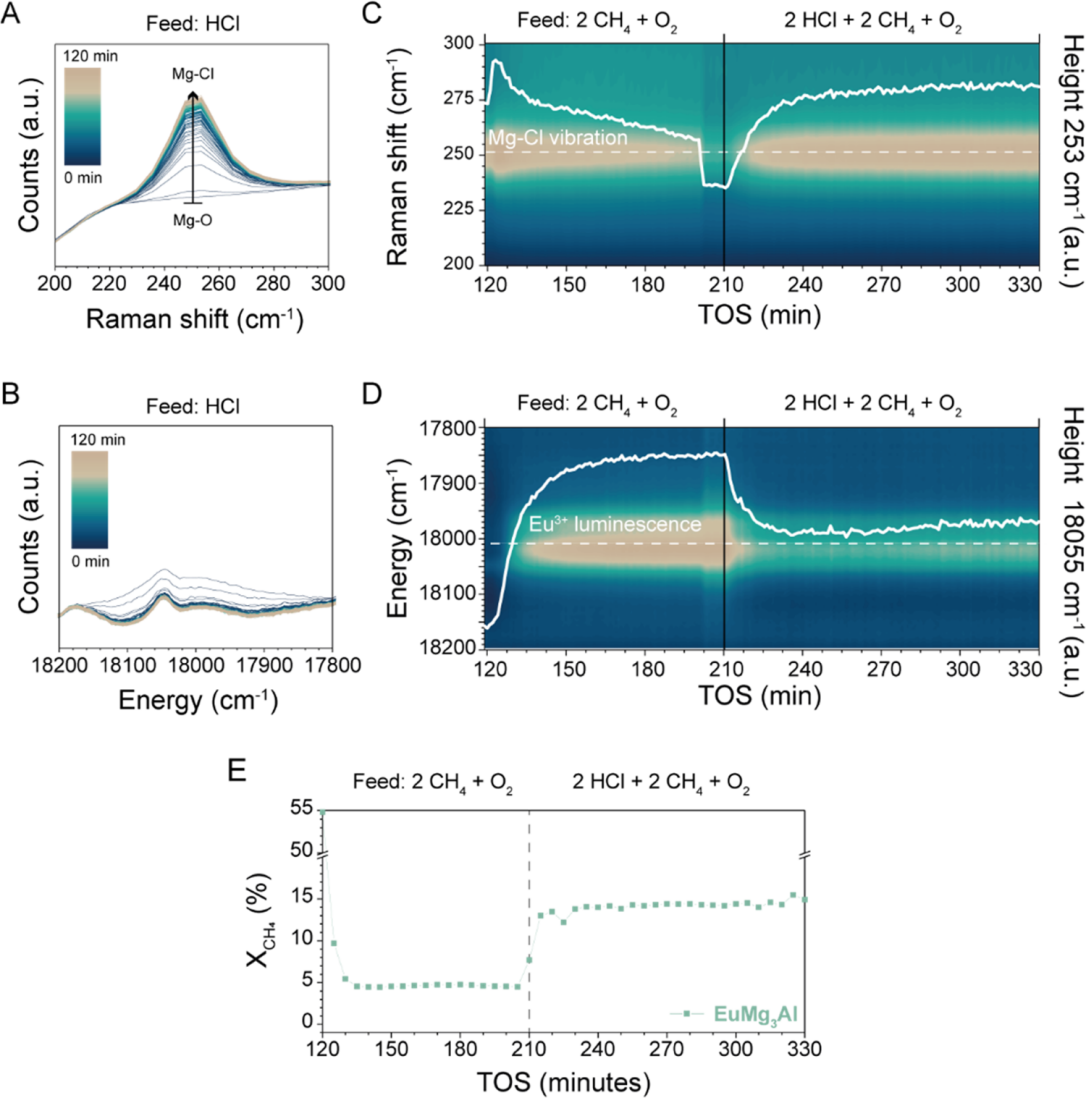
Operando Raman and luminescence measurements performed on EuMg_3_Al mixed-metal oxide (MMO) under MOC reaction conditions. (A) The Mg^2+^–Cl Raman vibration and the (B) ^5^D_1_ → ^7^F_2_ emission during the chlorination step. (C) Mg^2+^–Cl Raman vibration and the (D) ^5^D_1_ → ^7^F_2_ emission during the dechlorination and oxychlorination plotted as a function of time-on-stream (TOS). The height profiles of the Mg–Cl Raman vibration or the Eu^3+^ luminescence are given. (E) Methane conversion (*X*_CH_4__) plotted vs the TOS during the dechlorination and oxychlorination steps.

During the chlorination step, a significant increase in the signal of the Mg^2+^–Cl Raman vibration at 253 cm^–1^ was observable (Figure 8A). This observation is in line with the trends observed for MgO and Mg_3_Al. The luminescence signal from the as-synthesized catalyst was, however, hardly detectable and did not alter during the chlorination step (Figure 8B). The absence of a luminescence signal is most probably caused by the lack of symmetry of the Eu^3+^ center in the defective periclase-type structure.^48^ The chlorinated Eu^3+^ luminescence signal is also quenched at 500 °C; hence, the chlorination step was not observable.

Subsequently, the dechlorination step caused the catalyst to dechlorinate as the Mg–Cl vibration (Figure 8C) decreased in intensity and the Eu^3+^ luminescence signal increased (Figure 8D). The increase in luminescence can be explained by the fact that the material restructured during the chlorination reaction, enhancing the symmetry of the Eu^3+^ center. The subsequent dechlorination of the Eu^3+^ enhanced the radiative decay. Simultaneously, the *X*_CH_4__ revealed a fast discharge of chlorine, as indicated by the high initial *X*_CH_4__ of 55%, which quickly dropped to a steady-state *X*_CH_4__ of 5% (Figure 8E). This coincided with the fact that the Eu^3+^ signal increased quickly in the first 15 min of the dechlorination step (Figure 8D, 120–135 min). During the same period, the dechlorination of Mg^2+^ did show an increased rate but it was much less rapid than for Eu^3+^. A discrepancy in the dechlorination behavior was observed when comparing the Mg^2+^–Cl Raman and the Eu^3+^ luminescence, as Eu^3+^ approached a dechlorinated state already after 30 min while the Mg^2+^–Cl Raman steadily decreased. Lastly, during the oxychlorination step, both Mg^2+^ (Figure 8C) and Eu^3+^ (Figure 8D) were partially chlorinated, an indication that the addition of Mg^2+^ did not only chlorinate Al^3+^ but also facilitated the chlorination of Eu^3+^, which was shown to be rate-determining in MOC.^23^ We thus hypothesize that Mg^2+^ performed the same role as La^3+^ had in La_0.50_Eu_0.50_OCl. The high dispersion of Eu^3+^ throughout the material in combination with the chlorinating effect of Mg^2+^ makes EuMg_3_Al MMO to be highly active in the MOC reaction.

## 4 Conclusions

In this work, we demonstrate that nonreducible, Mg^2+^–Al^3+^ mixed-metal oxide (MMO) catalysts are very active for the methane oxychlorination (MOC) reaction, despite the low activity of the respective single-metal oxides MgO and γ-Al_2_O_3_. This was attributed to synergistic effects at play between Mg^2+^ and Al^3+^ in homogeneous mixtures, such as the synthesized MMO and MgAl_2_O_4_. To explain the observed synergy, based on thermodynamic calculations, catalytic results, and operando Raman spectroscopy observations, we propose that Mg^2+^ underwent chlorination under reaction conditions, acting as a chlorine buffer and chlorinating agent for Al^3+^, which was the active metal in the methane activation step.

The synthesized Mg_4_Al MMO possessed a high methane conversion rate of 4.63 mmol·g_cat_^–1^·h^–1^ and good stability over a 100 h period. However, the *S*_CO_ and *S*_CH_3_Cl_ could not meet the standard reported in the literature, as the *S*_CO_ > 40% and the *S*_CH_3_Cl_ < 50% at *X*_CH_4__ ∼ 10%. Hence, the MMO was functionalized with the redox-active Eu^3+^ by the partial replacement with Al^3+^, adding complementary Eu^3+^ properties to the Mg*_x_*Al MMO. For the 2 atom % of Eu-MMOs, the methane conversion rate was boosted to a maximum of 10.56 mmol·g_cat_^–1·^h^–1^, making these materials one of the most active catalysts reported. The redox-active Eu^3+^ made the catalyst active in the HCl oxidation reaction. Furthermore, the activity was preserved under high HCl concentrations in the feed, making the catalyst more resistant to HCl. The *S*_CO_ became tunable by varying the HCl concentration in the feed and could be suppressed by as much as 30%.

Even though Al^3+^ was needed for the activity in the reaction, the metal was also highly active in the catalytic destruction of polychlorinated C_1_, greatly influencing the activity–selectivity relation. Hence, the EuMg_3_Al MMO was not competitive in terms of selectivity to other benchmark catalysts reported in the literature. Lastly, combined operando Raman/luminescence spectroscopy revealed that the chlorination behavior of Mg^2+^ and Eu^3+^ was correlated, and we therefore hypothesize that Mg^2+^ also acted as a chlorinating agent for Eu^3+^. The high dispersity of Eu^3+^ throughout the material in combination with the chlorinating effect of Mg^2+^ makes the EuMg_3_Al MMO exhibit even greater activity in the MOC reaction than Mg_3_Al. The fact that a highly active catalyst material could be made of nonreducible Mg^2+^ and Al^3+^ sheds new light on the importance of synergistic effects in oxychlorination chemistry, while the addition of Eu^3+^ illustrates the importance of redox properties in improving the selectivity toward chlorinated methanes.

## Supporting Information Available

Lattice parameter calculations, conversion, yield, selectivity, and carbon balance calculations; characterization of reference materials; an explanation for the absence of postcharacterization; stability of γ-Al_2_O_3_; additional experimental data including catalytic data; thermodynamic equilibrium compositions; and Raman spectra (PDF)
