# Supplementary material for: Europium–Magnesium–Aluminum-Based Mixed-Metal Oxides as Highly Active Methane Oxychlorination Catalysts
Source: ACS Catal. 2023 Mar 30;13(8):5147–58. doi: 10.1021/acscatal.2c06344 (PMC10127201; doi:10.1021/acscatal.2c06344)
Supplement: Supplementary file 1 — cs2c06344_si_001.pdf [file cs2c06344_si_001.pdf]

Europium-Magnesium-Aluminum-based Mixed-Metal Oxides as Highly Active Methane Oxychlorination Catalysts

Bas Terlingen[#a], Jelle W. Bos[#a], Mathieu Ahr[§], Matteo Monai[#], Coert van Lare[§], Bert M. Weckhuysen[#]*

[#] Inorganic Chemistry and Catalysis group, Debye Institute for Nanomaterials Science and Institute for Sustainable and Circular Chemistry, Utrecht University, Universiteitsweg 99, 3584 CG Utrecht, The Netherlands; Email: B.M.Weckhuysen@uu.nl

[§] Nobian, Zutphenseweg 10, 7418 AJ Deventer, The Netherlands

[a] These authors contributed equally to this work.

1. Experimental Definitions and Calculations

1.1 Lattice Parameter Calculations

The lattice parameter a was calculated according to the following procedure and the results are summarized in Table S1. First, Origin 2017 multi peak fit tool was used to fit Voigt peaks functions, which in turn were used to determine the (200) x-ray diffraction (XRD) positions. From the peak position, the interplanar distance d (Å) was calculated according to Bragg's law

$$(Eq. S1)\ \lambda = 2d * \sin(\theta)$$

where λ and θ are the wavelength of the X-ray source (Å) and the angle of the incident light (˚) to the plane respectively. With the use of the interplanar distance and the Miller indices, the lattice parameters were then calculated. For the cubic MgO crystal system, the lattice parameter a (Å) can be calculated according to

$$(Eq. S2)\ \frac{1}{d^2} = \frac{(h^2 + k^2 + l^2)}{a^2}.$$

Table S1. The *2θ* of the (200) reflection, the interplanar distance *d* and the lattice parameter *a* are tabulated. The interplanar distance *d* and lattice parameter *a* are calculated according to (Eq. S1) and (Eq. S2), respectively.

Catalyst material	2θ (deg)	Interplanar distance *d* (Å)	Lattice parameter *a* (Å)
**MgO**	42,80	2.11	4.22
**$Mg_2AlO_{3.5}$**	43,37	2.08	4.17
**$Mg_3AlO_{4.5}$**	43,20	2.09	4.18
**$Mg_4AlO_{5.5}$**	43,08	2.10	4.19

**$Eu_{0.06}Mg_2Al_{0.94}O_{3.5}$**	43,32	2.09	4.17
**$Eu_{0.08}Mg_3Al_{0.92}O_{4.5}$**	43,07	2.10	4.20
**$Eu_{0.10}Mg_4Al_{0.90}O_{5.5}$**	43,12	2.10	4.19

1.2 Conversion, Yield, Selectivity and Carbon Balance

The CH_4 conversion, X_{CH4}, and O_2 conversion, X_{O2}, are calculated according to Eq. S3,

$$(Eq.\,S3)\ X_{a}(\%) = \frac{x_{a,\,inlet} - (x_{a,\,outlet} * ISCF)}{x_{a,\,inlet}} * 100\%$$

where $x_{a,\,inlet}$, $x_{a,\,outlet}$ and ISCF stand for the volumetric concentration of compound a at the inlet and outlet of the reactor and the Internal Standard Correction Factor, respectively. The yield of product i, Y_i, is calculated according to Eq. S4,

$$(Eq.\,S4)\ Y_i(\%) = \frac{x_i}{x_{CH4,\,inlet}} * 100\% * ISCF$$

where x_i and $ISCF$ stand for the volumetric concentration of carbon containing product I and the Internal Standard Correction Factor, respectively. The selectivity of product i, S_i, is calculated according to Eq. S5.

$$(Eq.\,S5)\ S_i(\%) = \frac{Y_i}{X_{CH4}} * 100\%$$

The CH_4 reaction rate is calculated according to Eq. S6,

$$(Eq.\,S6)\ R_{CH4}\left(\frac{mmol}{h * g_{cat}}\right) = \frac{P * F_T * x_{CH4,inlet} * \frac{X_{CH4}}{100}}{R * T * W_{cat}}$$

where P, F_T, R, T and W_{cat} stand for the pressure, total flow, gas constant, ambient temperature and catalyst weight. Finally, the carbon balance was calculated and measurements with their carbon balance > ± 5% were removed. The carbon balance was calculated according to Eq. S7.

$$(Eq.\ S7)\ Carbon\ balance = \sum Y(i) + 100 - X_{CH4}$$

2. Additional experimental data

2.1 Characterization of Reference Materials

An $Eu_{0.16}Mg_3Al_{0.84}O_{4.5}$, further denoted as $EuMg_3Al$-4%, and an $EuMg_3O_{4.5}$, further denoted as $EuMg_3$, MMO catalyst without any Al^{3+} were prepared according to the same synthesis procedure as described in section 2.1. Even though the desired Eu^{3+}:Mg^{2+}:Al^{3+} ratio and comparable S_{BET} and V_{pore} were obtained (Table S2), the synthesis yielded extra-framework phases (Figure S1).

Table S2. Overview of the physicochemical properties of the synthesized reference materials, including MgO, $Mg_3EuO_{4.5}$ and $Eu_{0.16}Mg_3Al_{0.84}O_{4.5}$. The BET surface area ($S_{BET}$), pore volume ($V_{pore}$) and the molar ratio as determined with ICP-OES is given.

Catalyst material	Further denoted as	N_2 Physisorption		Eu^{3+}:Mg^{2+}:Al^{3+} molar ratio (ICP-OES)
		S_{BET} (m^2/g)	V_{pore} (cm^3/g)	
**MgO**	MgO	167.9	0.51	-
**γ-Al_2O_3**	γ-Al_2O_3	261.5	0.85	-
**$EuMg_3O_{4.5}$**	Mg_3Eu	175.0	0.51	1.01:3:0
**$Eu_{0.16}Mg_3Al_{0.84}O_{4.5}$**	$EuMg_3Al$-4%	247.3	0.82	0.16:3:0.82
**$MgAl_2O_4$**	$MgAl_2O_4$	67.4	0.14	-

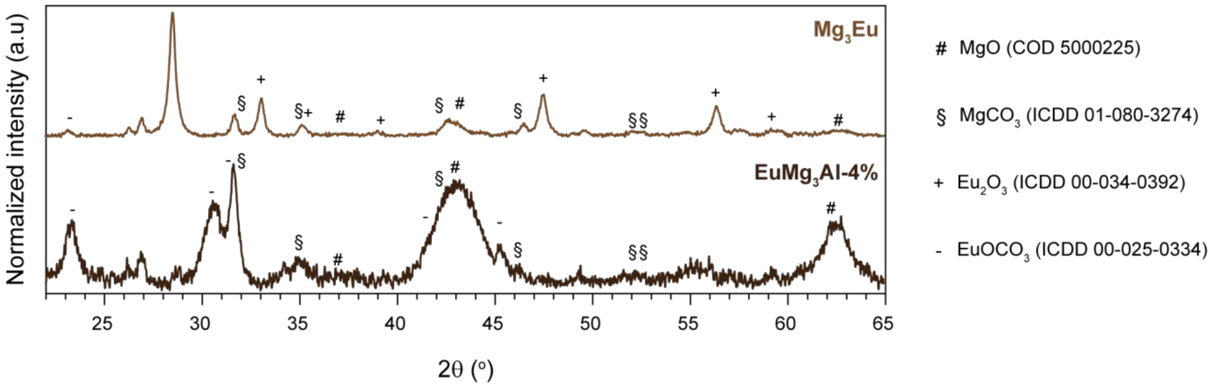

Figure S1. X-ray diffraction (XRD) patterns of the as-synthesized mixed-metal oxide (MMO) catalyst materials, including Mg_3Eu, $EuMg_3Al$-4% and $EuMg_3Al$-2%. With Eu^{3+} content above 2 atom%, extra framework phases are obtained.

2.2 Reliability of the post-characterization results

In this study, detailed post-characterization of the catalyst materials is hampered by the formation of hygroscopic metal chlorides. As $MgCl_2$ and $EuCl_3$ are formed in the reaction, exposure to water must be prevented in order to maintain the crystal structure and morphology of the spent catalysts, which poses a challenge with the applied set-up. Furthermore, the loosely bound Cl in the catalyst poses serious corrosion and contamination issues for certain characterization techniques, especially for techniques where the sample is either heated or treated in vacuum (e.g. sorption techniques). Hence, a dechlorination step is required in order to perform any post-characterization of the catalyst materials. Performing the dechlorination step unavoidably alters the structure of the catalyst. We therefore cannot guarantee that the dechlorinated catalyst is representative for the active catalyst in the reaction. Because of the abovementioned reasons, the post characterization results must be regarded as indicative to avoid any misconceptions on the reliability and reproducibility of this data.

2.3 Stability of γ-Al_2O_3

The γ-Al_2O_3 support material was subjected to 24 h of oxychlorination conditions to investigate whether the material possessed chemical, phase and structural stability. The surface area (S_{BET}), pore volume (V_{pore}) and catalyst weight before and after the reaction are tabulated in Table 1. The small change in catalyst mass between the Fresh and the Spent catalyst falls within the experimental error. No sign of material leaching was observed, which could be easily identified as quartz reactors were applied. Thermodynamic calculations (Figure 1B) support the experimental findings that the chlorination of Al_2O_3 to $AlCl_3$ is not occurring under reaction conditions.

Table S3. Overview of the physicochemical properties before and after exposure to 24 h of oxychlorination. The γ-Al_2O_3 was analyzed with N_2 physisorption for the change in surface area (S_{BET}) and pore volume (V_{pore}). Furthermore, the change in mass of the catalyst was also determined. Reaction conditions: CH_4:HCl:O_2:N_2:He of 2:2:1:1:14 (in mL/min), T = 450 ˚C, time on stream = 24 h.

Support material	S_{BET} (m^2/g)		V_{Pore} (cm^3/g)		Mass material (mg)			Phase change to
	Fresh	Spent	Fresh	Spent	Fresh	Spent	% change	
**γ-Al_2O_3**	261.5	244.6	0.85	0.86	149.7	150.5	+ 0.5%	None

2.4 Catalytic, Thermodynamic and Spectroscopic Data

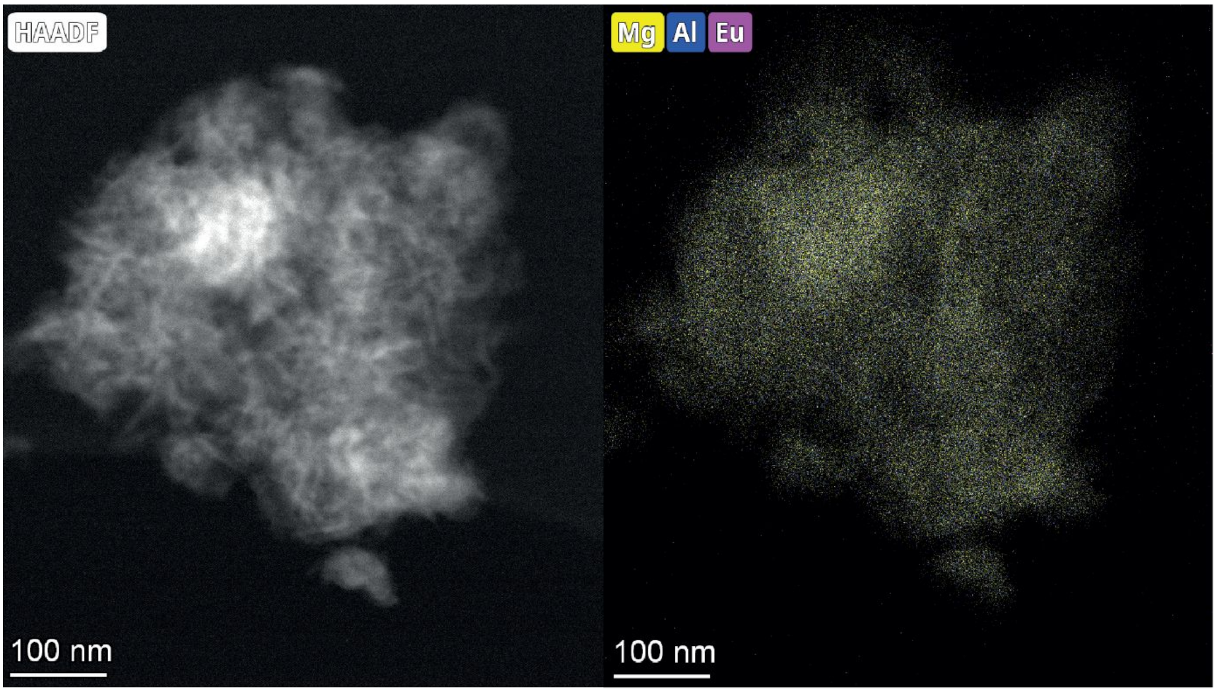

Figure S2. High-angle annular dark field-scanning transmission electron microscopy (HAADF-STEM) with energy dispersive x-ray spectroscopy (EDS) analysis revealed the uniform distribution on the nanoscale of Mg, Al and Eu of the MMO with 2 atom% Eu^{3+}.

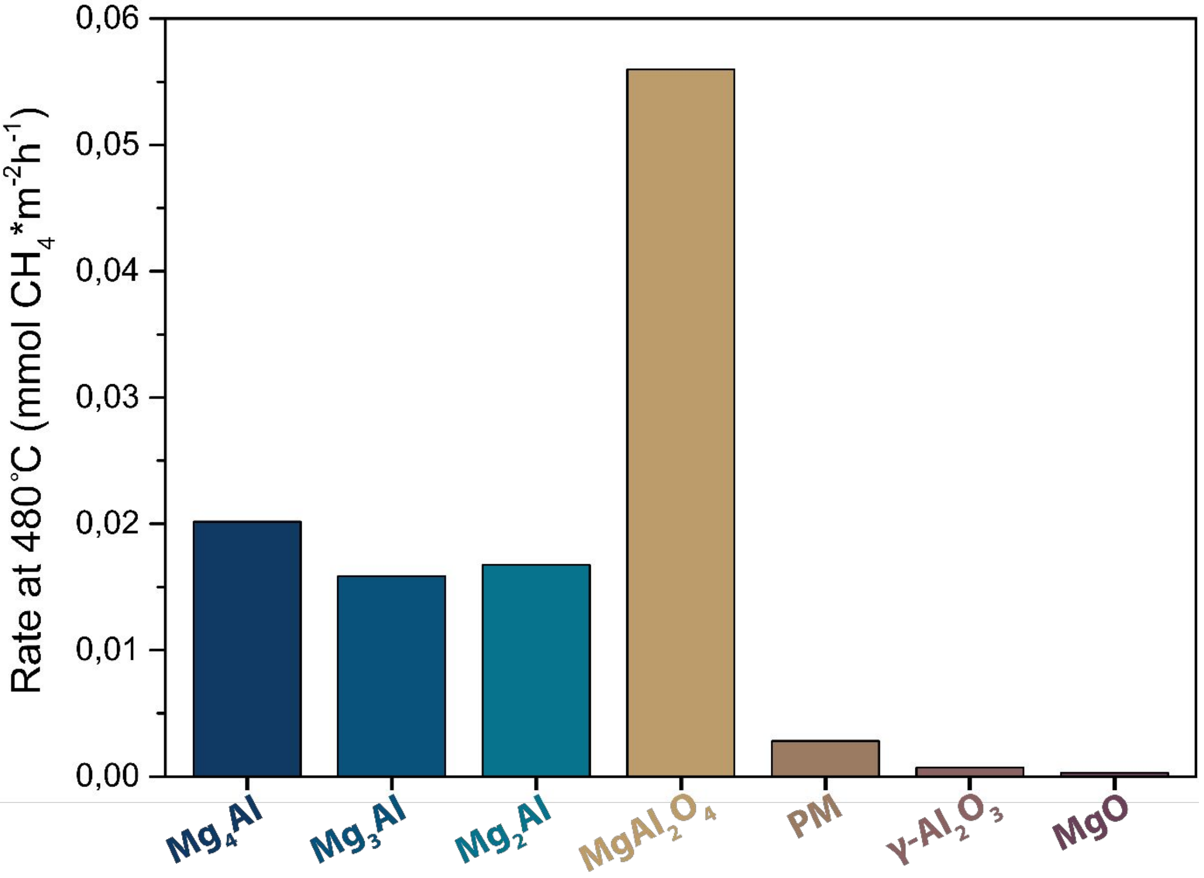

Figure S3. The CH_4 conversion rates of a selection of materials at 480 ˚C under 10% HCl normalized with respect to surface area

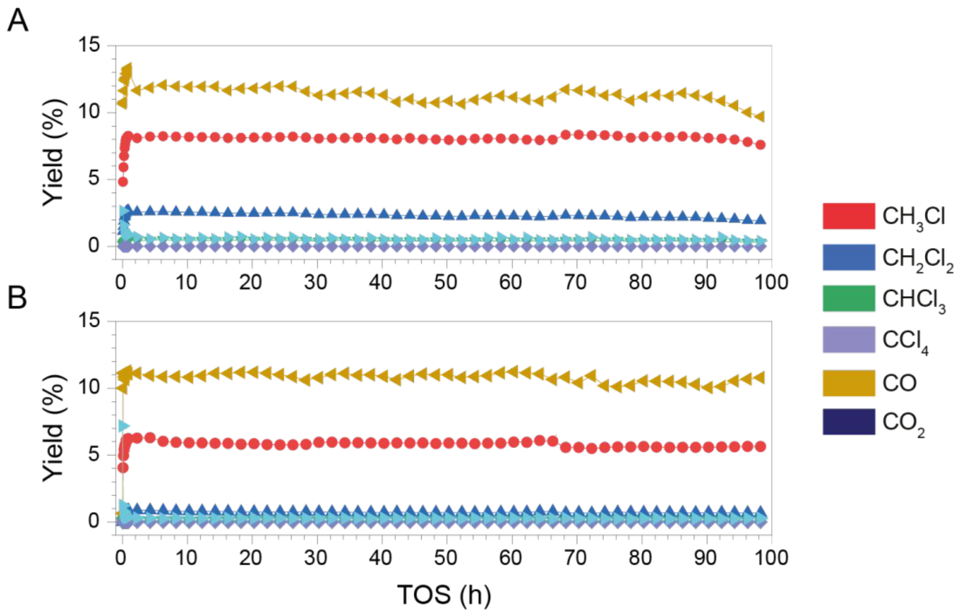

Figure S4. Catalytic stability of (A) Mg_4Al and (B) $EuMg_3Al$ in the methane oxychlorination (MOC) reaction tested for 100h time-on-stream (TOS). In both cases, the catalyst material produced a constant flow of products. A slight decrease in CO yield was observable for Mg_4Al after 90h TOS. As a higher W_{cat} was used for the stability test, the catalytic performance deviates from the results reported where a W_{cat} = 100 mg was used. Reaction conditions: CH_4:HCl:O_2:N_2:He of 2:2:1:1:14 (10% HCl, in mL/min), temperature 450 ˚C, W_{cat} = 250 mg.

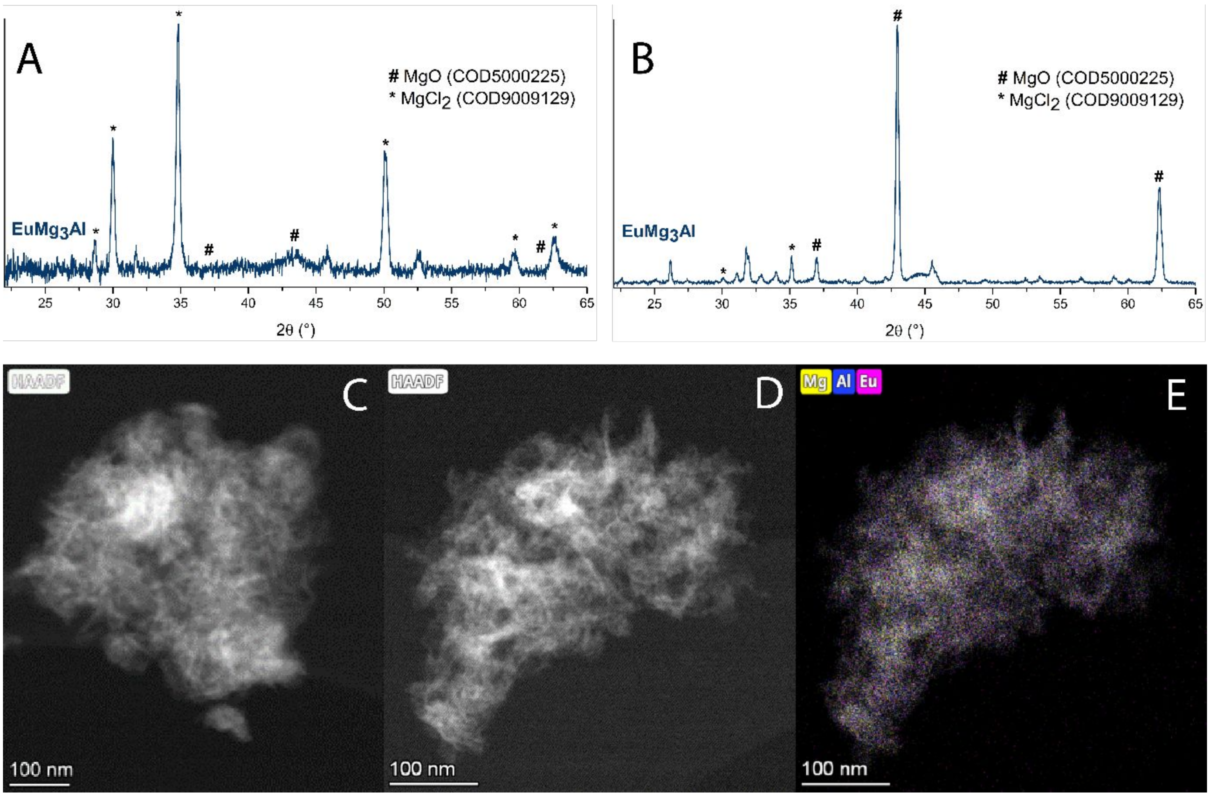

Figure S5. (A) X-ray diffraction (XRD) pattern of $EuMg_3Al$ after 24h of oxychlorination. Sample was transferred to the glovebox and the XRD was recorded under inert gas flow. The diffractions of the $MgCl_2$ are shifted slightly to higher 2Θ due to a higher bed-height of the powder sample. (B) X-ray diffraction (XRD) pattern of $EuMg_3Al$ after 24h of oxychlorination and subsequent dechlorination. (C) Transmission Electron Microscopy (TEM) image of the dechlorinated spent catalyst. (D,E) Scanning Transmission Electron Microscopy-Energy Dispersive x-ray Spectroscopy (STEM-EDS) does not show large agglomerates (Mg, Al and Eu) after 24h of reaction and

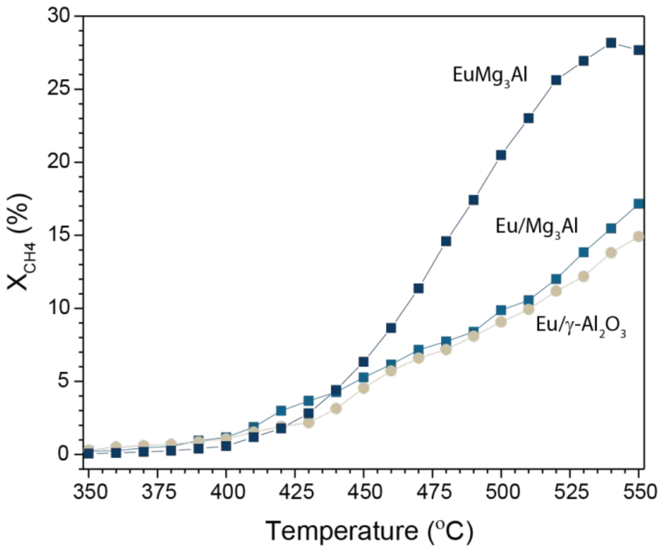

Figure S6. The CH_4 conversion (X_{CH4}) plotted versus the temperature under 10% HCl in the feed for $EuMg_3Al$, Eu/Mg_3Al and $Eu/\gamma\text{-}Al_2O_3$. Reaction conditions: CH_4:HCl:O_2:N_2:He of 2:2:1:1:14 (10% HCl, in mL/min) temperature ranging from 350 – 550 ˚C, W_{cat} = 100mg.

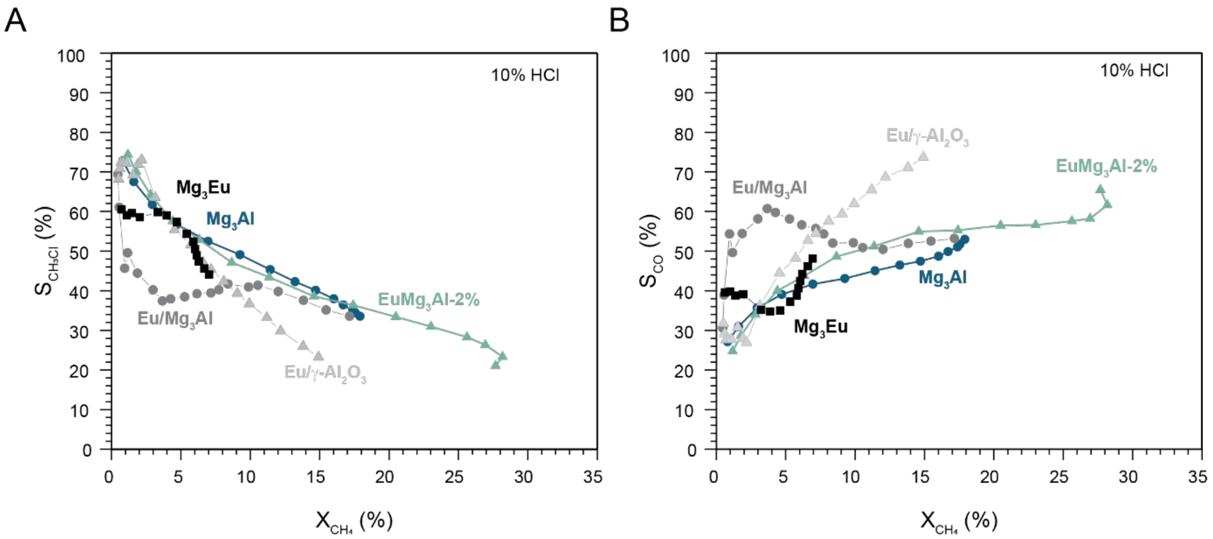

Figure S7. Non-isothermal activity-selectivity (X-S) relation for the Methane Oxychlorination (MOC) reaction plotted for Mg_3Eu, Mg_3Al, $EuMg_3Al$, Eu/γ-Al_2O_3 and Eu/Mg_3Al mixed-metal oxide catalysts under 10% HCl in the feed. The selectivity towards (A) CH_3Cl and (B) CO is given.

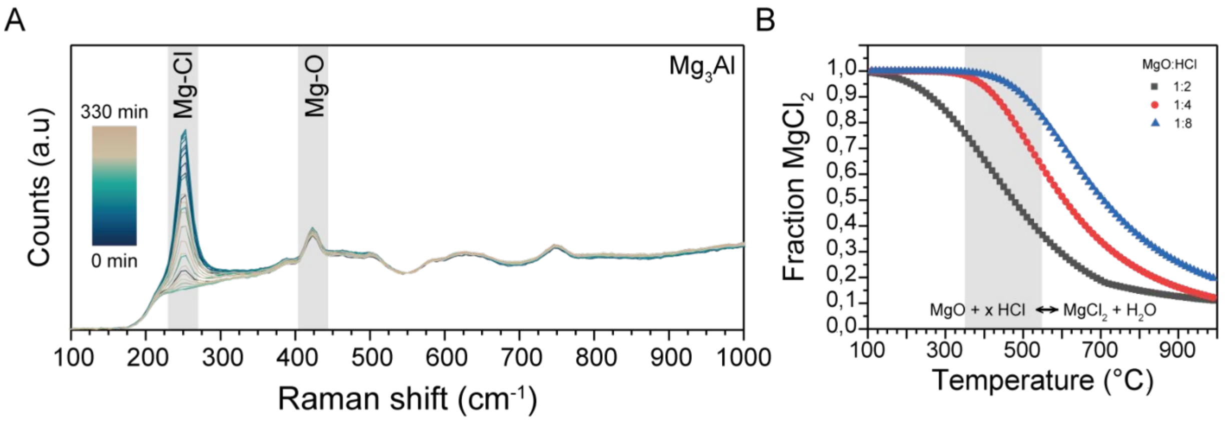

Figure S8. (A) Raman spectra of Mg_3Al corresponding to the chlorination-dechlorination-oxychlorination experiment presented in Figure 7. No spectral changes except for the appearance of the Mg-Cl vibration at 253 cm^{-1} were observed. (B) Thermodynamic equilibrium composition calculations revealed that an increment in the HCl concentration in the feed increases the fraction of Mg^{2+} that is chlorinated in the methane oxychlorination temperature range (grey box). Calculations were made with HSC Chemistry 9 equilibrium composition package from 100 - 1000˚C.

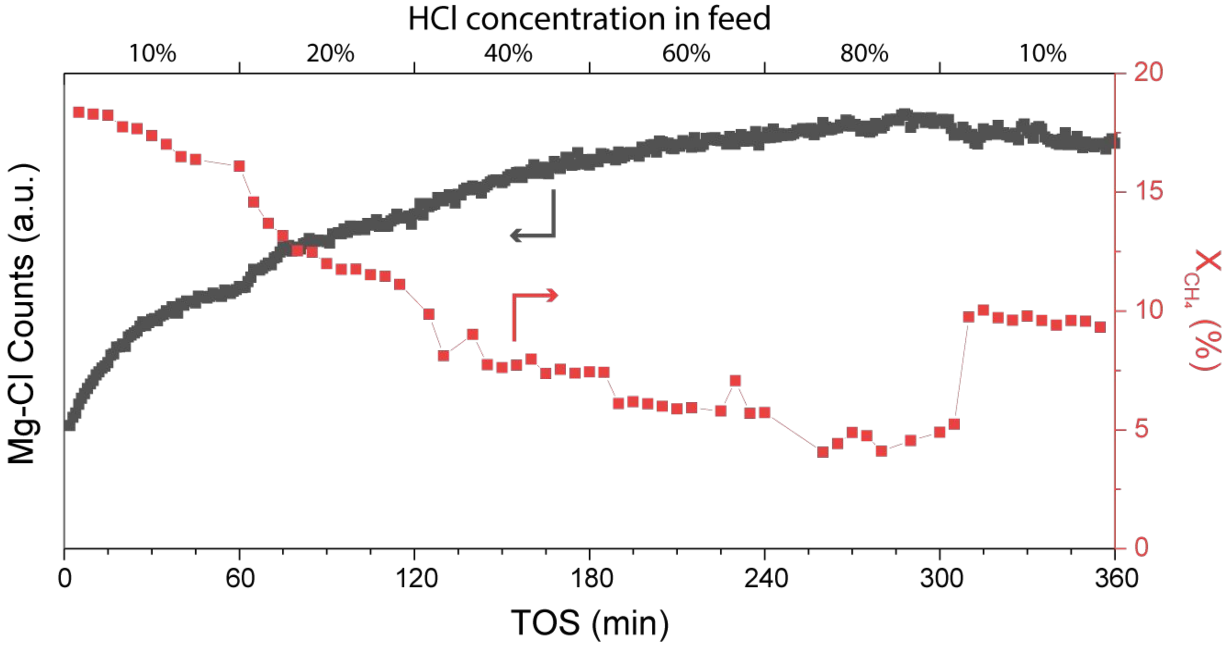

Figure S9. Influence of the HCl concentration in the feed on the Mg-Cl Raman intensity (left axis) and the methane conversion X_{CH4}, right axis) plotted versus the time-on-stream (TOS) for Mg_3Al. The HCl concentration was adjusted every 60 min.

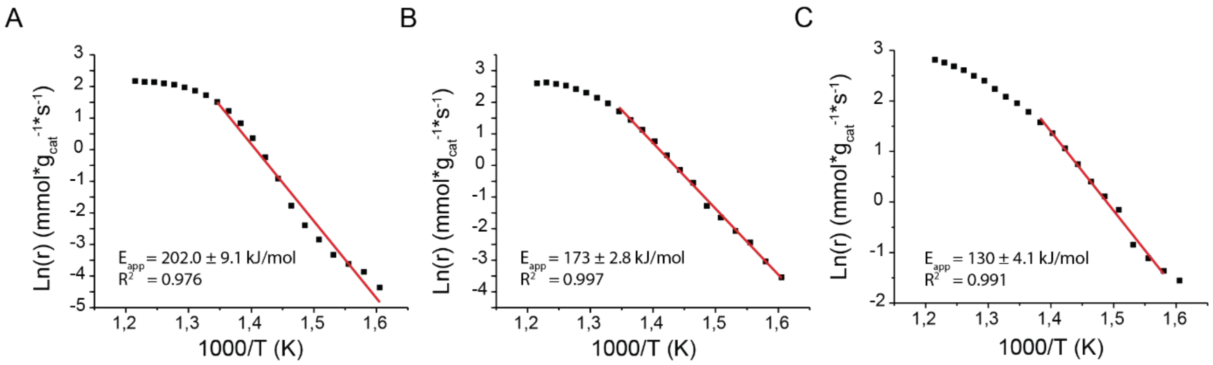

Figure S10. Apparent activation energy (E_{app}) of the methane oxychlorination (MOC) reaction over (A) Mg_3Al, (B) $EuMg_3Al$ and (C) CeO_2 using 10% HCl in the feed. Here, 100 mg of catalyst material (125–425 μm sieve fraction) was used to determine the apparent activation energy below 10% conversion to adhere to the requirements of the differential reaction model. Reaction conditions: CH_4:HCl:O_2:N_2:He of 2:2:1:1:14 (10% HCl, in mL/min), temperature ramped from 350-550 ˚C with steps of 10 ˚C and kept at every step for 45 minutes. The flattening of the curves at high temperature and conversion values is caused by mass transfer limitation, and by competition over the same active site for the catalytic destruction of chlorinated C_1 molecules.
